# Impact of Force Function Formulations on the Numerical Simulation of Centre-Based Models

**DOI:** 10.1007/s11538-020-00810-2

**Published:** 2020-10-06

**Authors:** Sonja Mathias, Adrien Coulier, Anass Bouchnita, Andreas Hellander

**Affiliations:** 1 Department of Information Technology, Uppsala University, Uppsala, Sweden; 2 Present Address: Ecole Centrale Casablanca, Bouskoura, Morocco

**Keywords:** Cell-based model, Force function, Numerical method, Monolayer growth

## Abstract

Centre-based or cell-centre models are a framework for the computational study of multicellular systems with widespread use in cancer modelling and computational developmental biology. At the core of these models are the numerical method used to update cell positions and the force functions that encode the pairwise mechanical interactions of cells. For the latter, there are multiple choices that could potentially affect both the biological behaviour captured, and the robustness and efficiency of simulation. For example, available open-source software implementations of centre-based models rely on different force functions for their default behaviour and it is not straightforward for a modeller to know if these are interchangeable. Our study addresses this problem and contributes to the understanding of the potential and limitations of three popular force functions from a numerical perspective. We show empirically that choosing the force parameters such that the relaxation time for two cells after cell division is consistent between different force functions results in good agreement of the population radius of a two-dimensional monolayer relaxing mechanically after intense cell proliferation. Furthermore, we report that numerical stability is not sufficient to prevent unphysical cell trajectories following cell division, and consequently, that too large time steps can cause geometrical differences at the population level.

## Introduction

Discrete cell-based models are becoming increasingly popular for simulating tissue mechanics. In contrast to continuum models that average over the cell density, cell-based models represent each cell individually. Therefore, they readily allow for incorporating cellular events such as cell division, cell differentiation or cell death, as well as cell heterogeneity across a population. As a result, these models have been used to probe biological questions of how the interplay of individual cell behaviour affects population-level measures (e.g. (Meineke et al. 2001; Li and Lowengrub 2014; Kursawe et al. 2015; Atwell et al. 2015)).

There are different kinds of cell-based models that can be divided into two general categories. The first category, so-called *on-lattice* models, restricts the movement of the cells to a grid. Cellular automata (Peirce et al. 2004) and cellular Potts (Graner and Glazier 1992) models are examples. In cellular automata models, cells are typically restricted to occupy a single lattice site and move between lattice sites according to a fixed set of rules. In contrast, in cellular Potts models cells are composed of multiple lattice sites, enabling the cell shape to be resolved more realistically. The whole system explores the energy landscape using a Metropolis–Hastings approach. One drawback of on-lattice models is that they can exhibit grid-related artefacts on structured meshes due to the directional restriction, e.g. cells can only “push” neighbours along fixed axes as defined by the underlying grid (Van Liedekerke et al. 2015; Drasdo et al. 2018).

The second category, *off-lattice* models, are continuous in space and hence circumvent this issue. Again they vary with respect to how detailed the cell shape is modelled. Centre-based models (CBMs)—also referred to as cell-centre models—track the cell midpoints over time as cells interact mechanically according to pairwise spring-like forces (Meineke et al. 2001; Drasdo and Hoehme 2005). In this model, cells are either represented as overlapping spheres (OS variant), or using a Voronoi tessellation (Voronoi variant). Vertex models (Fletcher et al. 2014), on the other hand, discretize the cell boundary instead and evolve the tissue according to interfacial tension and pressure within the cells. As a result, they can be applied to study complex cellular behaviour such as cell growth, stretching and deformation (Tamulonis et al. 2011). At an even higher level of detail and correspondingly higher computational cost, there are the immersed boundary method (Rejniak 2007) and the subcellular element method (Newman 2007).

Discrete cell-based models—independent of being on- or off-lattice—can be coupled to PDE models for simulating the concentration of chemical compounds in the cellular environment or even an ODE model for simulating intracellular dynamics (Cilfone et al. 2015; Macklin et al. 2016; Ward et al. 2020). An extensive review of cell-based models for general tissue mechanics can be found in Van Liedekerke et al. (2015). Additionally, there are several reviews dealing with prominent applications areas, such as tumour growth (Rejniak and Anderson 2010; Metzcar et al. 2019) and morphogenetic problems (Glen et al. 2019; Fletcher et al. 2017; Tanaka 2015). In Osborne et al. (2017), the authors compare five cell-based frameworks (cellular automata, cellular potts, CBM OS and Voronoi variants and vertex models) with respect to four common biological problems: cell sorting, monoclonal conversion, lateral inihibition and morphogen-dependent proliferation. They conclude that each model has its preferred application for the study of which it was originally designed, but that most models can be adapted for all applications with varying effort and computational cost.

In this study, we focus on the centre-based model, in particular the OS variant, to which we will from now on refer to as CBM or CBM OS when we want to stress particularities about the latter. CBMs have been successfully applied to a large variety of biological problems ranging from the simulation of monolayer and spheroid growth (Drasdo and Hoehme 2005; Galle et al. 2006) to the cellular reorganization in the intestinal crypt (Meineke et al. 2001). See Van Liedekerke et al. (2018) for a recent overview. There exist multiple simulation frameworks that implement CBMs, several of which are open source. All of them tailor to specific needs, but allow for modelling the core features of CBMs. *Chaste* is a multi-purpose framework implementing several cell-based models and CBMs in particular (Cooper et al. 2020; Mirams et al. 2013; Pitt-Francis et al. 2009). *MecaGen* is a framework focusing on the coupling between cell mechanics and gene regulatory networks (Delile et al. 2017). Most recently, *PhysiCell* was released in 2018 (Ghaffarizadeh et al. 2018). It aims to simulate up to a million cells and has been used mainly to model breast cancer (Macklin et al. 2009, 2012; Hyun and Macklin 2013). Moreover, there exist the closed-source frameworks *CellSys* (Hoehme and Drasdo 2010b), *EPISIM* (Sütterlin et al. 2013) and *Biocellion* (Kang et al. 2014).

In general, one can observe the trend of simulating an ever-growing number of cells in order to realistically describe biological systems. The efficiency of simulations is critical to this endeavour. Highly efficient schemes not only allow for the simulation of large-scale systems, but also make systematic inference and model exploration feasible. Here, the CBM framework, and in particular the OS variant in its simplest form, strikes an attractive balance between numerical efficiency and the capability to incorporate cell mechanics and biophysical measurements. The biophysical basis of cell–cell interactions has been discussed extensively in prior work (Drasdo and Hoehme 2005; Drasdo 2007; Drasdo et al. 2007), where the Johnson–Kendall–Roberts (JKR) and extended Hertz theories can provide descriptions founded in physics (Byrne and Drasdo 2008). However, these force descriptions are computationally expensive to evaluate, and in practice other, simpler functional forms are considered.

Motivated by the need for robust and efficient simulations, this study focuses on the complementary question of comparing those force functions used in open-source frameworks for CBMs. More specifically, we choose the functions used in Chaste, PhysiCell and MecaGen (Cooper et al. 2020; Ghaffarizadeh et al. 2018; Delile et al. 2017). Given the relatively few numbers of free parameters in these force functions, we first ask if there are systematic ways to parameterize them such that they result in indistinguishable macroscopic output for a biologically realistic test problem. We show that it is sufficient to accurately match the relaxation time of cells after division in order to observe good agreement of trajectories for the population radius of a monolayer relaxing mechanically in two dimensions after a period of intense proliferation. Based on such a parameterization, we then ask if there are significant advantages and disadvantages of different force functions from a numerical robustness- and efficiency point of view.

Different solution strategies and integration schemes for Vertex Models were discussed and evaluated in detail in Kursawe et al. (2017). However, to the best of our knowledge a systematic comparison of different commonly used schemes for CBMs has not been published. The overwhelming majority of CBM implementations use the forward Euler method for solving the coupled ODE system for centre positions (Cooper et al. 2020; Delile et al. 2017; Hoehme and Drasdo 2010a), with PhysiCell being a notable exception in that it uses the second-order Adams–Bashforth method (Ghaffarizadeh et al. 2018). Pathmanathan et al. (2009) study mechanical properties of a tissue simulated with CBMs—both the OS and the Voronoi variants—subject to compression, tension and shear under a linear spring force and two physics-based forces, Hertz and Johnson–Kendall–Roberts. Instead of probing the mechanical properties of a non-proliferating tissue, we are concerned with the interplay between cell proliferation and the impact of the resulting strong, highly localised forces on the mechanical relaxation of a growing population. Here, a critical aspect is the potentially large stiffness caused by highly compressed cells right after cell division. A key contribution of this paper is a comparison of integration schemes for this setting, taking into account the dependence on the force functions for stability and error.

For simplicity and easier visualization, we restrict ourselves to two-dimensional test problems. However, it should be noted that due to the simple geometrical representation of cells in the CBM OS framework as spheres, our study could be readily extended to three dimensions.

This article is structured as follows. In Sect. 2, we review the key numerical aspects of CBMs. In Sect. 3, we describe three force functions encountered in popular simulation frameworks and compare their qualitative behaviour for a fixed parameterization. In Sect. 4, we study their numerical properties with regards to numerical stability and accuracy in combination with first- and second-order solvers. Lastly, we discuss our findings and conclusions in Sect. 5.

## Implementation of Centre-Based Models

**Fig. 1:**
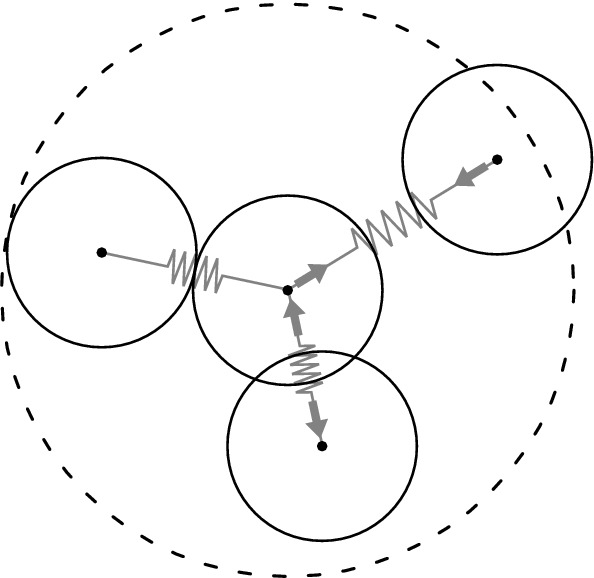
Illustration of the main idea of centre-based models, in particular the OS variant. Cell midpoint positions are tracked and the shape is assumed to be spherical, potentially overlapping with its neighbours. Cells move according to pairwise mechanical interactions, which can be repulsive (when overlapping) or adhesive (when non-overlapping but close). We assume that mechanical interactions are limited within a fixed distance from the cell centre, represented here by the dashed circle. All cells within this radius are assumed to interact with the cell in the centre

In this section, we review the key numerical assumptions made when implementing CBMs, namely (i) the update equation for cell positions, (ii) the numerical solution of this equation, (iii) the way cell connectivity is defined and updated, (iv) how cell-level events are incorporated into the cell mechanics simulation and (v) how the cell cycle and cell division events are modelled in particular. In literature and existing software, there is a considerable variation when it comes to the details of the implementations, depending on the biophysiology studied and the computational budget. Motivated by the need to simulate increasingly large systems, we consider the simplest and computationally cheapest implementations as these tend to be the most scalable. For this reason, we focus on the OS variant of CBMs, where cell shape is not explicitly resolved but cells are instead modelled as spheres which can potentially overlap. Figure 1 illustrates the main idea. As depicted, cell connectivity is defined using a simple maximum interaction radius.

Although several of the available frameworks for simulating CBMs are open and extensible, their large feature set, their focus on supporting modellers and corresponding code complexity make them less suitable for rapid experimentation with numerical parameters. Here, we study relatively simple models of cell mechanics with the need for full control of all numerical settings. Therefore, we developed our own centre-based code in Python to ensure consistency across the numerical experiments. To facilitate reproducibility and in the hope that it will be useful for others for similar studies, the software is publicly available at: https://github.com/somathias/cbmos.git. This repository contains the CBMOS software, as well as all Jupyter Notebooks used to generate the plots presented in this manuscript. Unit tests used throughout the development of this software are also included in this repository. (Note that we use CBMOS as the name of our simulation code, whereas the spelling “CBM OS” refers to the general framework of the overlapping spheres variant of the centre-based model.) The following subsections detail the implementation assumptions we consider in this study.

### Update Equation for the Cell Positions

Let $\mathbf{x} _i$ denote the midpoint coordinates of the $i$th cell and $m_i$ its mass. Applying Newton’s second law to the cell movement we obtain

1 $$\begin{aligned} m_i \mathbf{a} _i = m_i \ddot{\mathbf{x }}_i = \mathbf{F} _i, \end{aligned}$$

where $\mathbf{a} _i$ refers to the acceleration of cell $i$ and $\mathbf{F} _i$ to the force acting on it. We assume that $\mathbf{F} _i$ consists of two types of forces. First, a drag force $\mathbf{F} ^{\text {drag}}_i$ stemming from the cell’s friction with the viscous environment is directed against the cell’s direction of motion. This force is proportional to the cell’s velocity,

2 $$\begin{aligned} \mathbf{F} ^{\text {drag}}_i = - \eta \mathbf{v} _i = - \eta \dot{\mathbf{x }}_i. \end{aligned}$$

Here, $\eta$ denotes the drag coefficient. Secondly, neighbouring cells exert mechanical forces on the cell. In the simplest case these involve repulsive forces due to limited cell compressibility, but they usually also include cell–cell adhesion. Interactions are assumed to be pairwise and symmetric, i.e.

3 $$\begin{aligned} \mathbf{F} ^{\text {cells}}_i = \sum _{j \ne i} \mathbf{F} _{ij}, \end{aligned}$$

where the sum runs over all neighbours, excluding the cell itself. Which cells are considered neighbours depends on the definition of cell–cell connectivity, see Sect. 2.3 below for details. The resulting governing equation for the cell midpoint motion reads

4 $$\begin{aligned} m_i \ddot{\mathbf{x }}_i = - \eta \dot{\mathbf{x }}_i + \sum _{j \ne i} \mathbf{F} _{ij}. \end{aligned}$$

This is a system of second-order ordinary differential equations governing the cell centre positions, with one equation for each degree of freedom. As the microenvironment for the cells has a very low Reynolds number (the Reynolds number describes the ratio of inertial to viscous forces) (Purcell 1977), inertial effects such as acceleration are neglected. This commonly applied “inertialess” assumption, $m_i \ddot{\mathbf{x }}_i \approx 0$, reduces the update equation to the system,

5 $$\begin{aligned} \dot{\mathbf{x }}_i = \frac{1}{\eta } \sum _{j\ne i} \mathbf{F} _{ij}. \end{aligned}$$

Note that the drag coefficient $\eta$ effectively acts as a scaling of the pairwise force contributions $\mathbf{F} _{ij}$. This allows us to (arbitrarily) fix it as $\eta =1$.

For increasing levels of physical realism in the simulations, Eq. (5) might be complemented with additional terms representing friction between cells, repulsive and adhesive interaction between the cell and the extracellular matrix and cell migratory behaviour (Drasdo and Hoehme 2005; Van Liedekerke et al. 2015). The force-based formulation considered here has become the standard way of implementing CBMs and is favoured over energy-based formulations (numerically studied using Monte Carlo methods such as the Metropolis algorithm (Drasdo et al. 1995; Byrne and Drasdo 2008)) due to the straightforward interpretation of the time scale and a more intuitive way of treating custom cell interactions (Van Liedekerke et al. 2015).

### Numerical Methods for Solving the Update Equation

For an initial value problem stated as

6 $$\begin{aligned} \dot{y}= f(t,y) \text {, } y(t_0) = y_0, \end{aligned}$$

a numerical scheme provides an approximation for function values $y_n \approx y(t_n)$ at discrete time points $t_n$, $n=1,...,N$. The simplest numerical scheme, the forward Euler method, calculates the next function value by taking a step in the direction of the current gradient (Griffiths and Higham 2010),

7 $$\begin{aligned} y^{\text {Euler}}_{n+1} = y_n + \varDelta t \, f(t_n, y_n), \end{aligned}$$

where $\varDelta t$ is the step size. If the step size is chosen constant over the time interval $t_0$ to $t_N$, then $\varDelta t = (t_N-t_0)/N$. The forward Euler method is a first-order scheme, meaning that as long as $\varDelta t$ is sufficiently small, the local error in one single time step is proportional to $\varDelta t^2$ and the global approximation error at $t_N$ thus proportional to $\varDelta t$. Roughly speaking, halving the step size for a first-order scheme makes the solution twice as accurate.

Higher-order schemes improve the convergence rate at the cost of additional function evaluations. An example is the midpoint rule (Chapra 2012),

8 $$\begin{aligned} y^{\text {midpoint}}_{n+1} = y_n + \varDelta t f\left( t_n+\frac{\varDelta t}{2},y_n+\frac{\varDelta t}{2}f(t_n, y_n)\right) . \end{aligned}$$

The midpoint rule is a second-order method, which means that halving the step size divides the error by four. Hence, fewer steps are needed to achieve a given accuracy compared to the forward Euler method, although each single step will be more costly.

Both methods are one-step methods, meaning that they calculate the function value at the next time point based on the current function value only. Multi-step methods additionally take past function values into account. One of the simplest two-step methods is the Adams–Bashforth method (Griffiths and Higham 2010),

9 $$\begin{aligned} y^{\text {Adams-B.}}_{n+1} = y_{n} + \tfrac{3}{2} \varDelta t f(t_{n},y_{n}) - \tfrac{1}{2} \varDelta t f(t_{n-1},y_{n-1}). \end{aligned}$$

In addition to the order of the scheme, the schemes’ stability is an important characteristic. A numerically unstable solution will oscillate and grow without bounds, even though the true solution does not. In this case, the step size needs to be reduced to recover a stable, bounded solution. The stability region depends on both the numerical scheme and on the ODE problem to be solved, and this imposes an upper bound on $\varDelta t$ for which the scheme can be used. The forward Euler method has very poor stability properties and may hence require very small time steps.

### Definition of Cell Connectivity

The right-hand side in Eq. (5) involves the evaluation of pairwise interaction forces for all cells $j=1,\ldots , M$ in a neighbourhood of the cell with midpoint $\mathbf{x} _i$. In the overlapping spheres variant of CBMs that we consider, cell connectivity, i.e. which cells are allowed to mechanically interact, is defined via a maximum interaction distance (see Fig. 1). This can be efficiently implemented in two and three dimensions. For a large number of cells, the naive implementation of checking all $K^2$ pairwise interactions in a system with a total number of $K$ cells can be improved by introducing so-called bounding boxes that keep track of closely located cells (Hockney and Eastwood 1988). In the absence of these boxes, the computation of pairwise forces is inefficient because most of the distances between cells are larger than the maximum interaction distance. Indeed, for large systems, one cell is in contact with a small number of other cells compared to the total number of cells. Therefore, dividing the computational domain into boxes significantly lowers the computational cost of numerical simulations at the expense of a more complex simulation algorithm that needs to keep the data structure up-to-date. For the present study, we have chosen to not implement bounding boxes since they affect the performance per time step, while we are interested in how force functions and numerical methods affect the total number of time steps in a given simulation.

It is well known that defining cell connectivity using only distance information between cells can lead to the problem of collapsing volumes (Pathmanathan et al. 2009). In a densely packed population, newly divided cells can potentially interact with their next-to-nearest neighbours, resulting in the adhesive forces exerted by their (many) next-to-nearest neighbours becoming larger than the repulsive forces exerted by their (few) direct neighbours and thus compressing the overall volume of the population. Different force functions show a varying sensitivity to this issue, the linear spring being particularly problematic (Pathmanathan et al. 2009).

It is also possible to take topological information into account to ensure that cells only interact with neighbours with which they are in direct contact, i.e. that no other cell is located between them. This is done in the Voronoi variant of the CBMs using a Voronoi tesselation of the cell midpoints where cells are considered in mechanical contact if they share an edge in the associated Delaunay triangulation (Meineke et al. 2001; Schaller and Meyer-Hermann 2005; Meyer-Hermann 2008; Kennedy et al. 2016). The Voronoi polygons can then also be used to estimate cell shape and volume. This approach makes the Voronoi variant-CBM more robust to the issue of collapsing volumes at an increased computational cost compared to the OS variant (Osborne et al. 2017; Pathmanathan et al. 2009).

### Time-Driven Versus Event-Driven Simulation

In addition to cell mechanics, a CBM simulation usually consists of a number of cell events, such as cell division, cell death and potentially subcellular events such as a transition in cell cycle models. Typically, simulations proceed through a split-step scheme with fixed time steps for each main model feature (mechanics, molecular diffusion, cell events etc.)(Ghaffarizadeh et al. 2018). This time-driven approach is a good way of handling systems with different physics and with different timescales, but it also introduces an additional level of approximation due to the decoupling of terms during the time interval that the system is updated with some of the physics frozen—a splitting error. This error is, in general, hard to estimate.

As an alternative, event-driven simulation methods maintain a list of time points at which events affecting the system will take place (Meyer 2014). In the context of the simulation of cellular systems, this allows to simulate the mechanics until the next event time, apply the corresponding cellular event to the cell population and then continue with simulating the mechanics. This is possible as long as events can be scheduled in advance, e.g. by drawing a division time from a suitable distribution at cell birth, and do not depend on the current cell or population state. We chose this latter approach since it accurately resolves the cellular dynamics of the population for our model problems.

### Cell Cycle Model and Cell Division Implementation

In general, CBMs employ cell-cycle models of varying complexity to model the proliferation of cells, ranging from choosing a uniformly distributed cell cycle duration to nutrient- or contact-based cell cycle models (Meineke et al. 2001; Drasdo and Hoehme 2005; Macklin et al. 2012; Atwell et al. 2015). Furthermore, there exist different algorithms for implementing the cell division process itself. Biologically, this process involves the formation of a contractile actin ring at the division plane of the mother cell. As this ring contracts, the cell is gradually deformed leading to the formation of two daughter cells that eventually pinch off into two adhering cells of roughly half the volume of the mother cell. Since cell shapes are not explicitly modelled in the CBM, the details of this process cannot be captured directly. Instead, different types of approximations are employed. A thorough discussion on the various aspects of cell division algorithms, including a summary of additional commonly modelled mechanisms in the cell cycle such as contact inhibition and apoptosis, can be found in Van Liedekerke et al. (2018). We here briefly discuss two approaches.

In Drasdo and Hoehme (2005), two main phases of division are recognized. A cell entering the cell cycle first shows spherical growth. Then, when it splits into two daughter cells, they are treated as a dumbell in which the cells gradually separate by increasing the distance to each other. Alternatively, cells are treated as a dumbell directly when they enter the cell cycle and spherical growth and separation happens simultaneously (Hoehme and Drasdo 2010a). Dumbell formation, although somewhat close to the biological processes in concept, introduces additional complexity in the model due to the breaking of spherical symmetry (Drasdo and Hoehme 2005).

A simpler and more commonly used algorithm is to instantaneously place two newly formed daughter cells of a smaller radius close to each other (with an overlap) in the space previously filled by the mother cell (Schaller and Meyer-Hermann 2005). The cells will then relax into mechanical equilibrium with their surroundings according to the specific force function dynamics used in the simulation, as well as increase their radius to the size of the former mother cell. This, or close variants of it, is also the algorithm used in several recent high-performance and parallel implementations (Harvey et al. 2015; Ghaffarizadeh et al. 2018; Cytowski and Szymańska 2014). Implementation-wise this is a very simple and efficient algorithm. However, it may lead to locally and transiently high, unphysical force values right after cell division (Schaller and Meyer-Hermann 2005; Van Liedekerke et al. 2018). This can be seen as a consequence of the fundamental model assumption underlying the CBM, i.e. the assumption of pairwise cell interactions not being valid in highly compressed scenarios (Van Liedekerke et al. 2018). In Van Liedekerke et al. (2019) a multiscale approach based on deformable cell model simulations is used to correct forces for highly compressed structures. We will instead investigate how the population level behaviour is affected if these high local forces are not correctly resolved numerically.

**Fig. 2:**
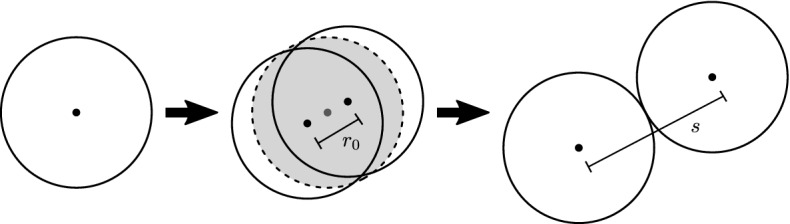
Illustration of the cell division steps in our implementation. Left: A cell ready to proliferate. Middle: Placement of two daughter cells of radius equal to that of the mother cell. The former position of the mother cell is the shaded region. The distance between the centres of the daughter cells is the initial separation $r_0$. The cell division direction is chosen randomly. Right: After mechanical relaxation, cell midpoints are separated by the rest length *s*

For our numerical study, we consider only cell division events and do not model the cell cycle explicitly. We adopt the simplified division algorithm detailed above and in Schaller and Meyer-Hermann (2005), but without accounting for cell growth. At the beginning of each simulation, we let cells divide instantaneously into two overlapping daughter cells of volume equal to the mother cell, see Fig. 2. No further subsequent cell division events take place in our numerical experiments. Note that our simulation framework alternatively allows for drawing the cell cycle duration according to some suitable random distribution (e.g. the Erlang distribution as suggested in (Chao et al. 2019)). Furthermore, other simulation frameworks such as PhysiCell and Chaste use more complex models for the cell cycle (Ghaffarizadeh et al. 2018; Cooper et al. 2020). However, we restrict the present study to a simple fixed cell cycle implementation specifically tailored to our numerical experiments to be able to more easily interpret the obtained results.

The cell division direction is chosen randomly according to a uniform distribution of the angle. This is in good agreement with experimental data showing that cell division is oriented towards a random direction in the absence of external signals (Siegrist and Doe 2006).

## Cell–Cell Interaction Forces

The cell–cell interaction force $\mathbf {F}_{ij}$ in Eq. (5) describes how cells interact mechanically and thus determines the biophysical behaviour the model can capture. In this study, we consider force functions based on the following assumptions:

1. The pairwise force can be written as ${\mathbf{F}}_{ij} = F(\Vert {\mathbf{r}}_{ij}\Vert ) \frac{{\mathbf{r}}_{ij}}{\Vert {\mathbf{r}}_{ij} \Vert }$, where ${\mathbf{r}}_{ij}={\mathbf{x}}_{j}-{\mathbf{x}}_{i}$ and $F$ is a scalar force. This means the force acts only in direction of the vector between cell centres and its magnitude depends only on their distance.
2. There exists a distance between cell midpoints (the rest length $s$) at which cells are in equilibrium, i.e. they exert no forces on each other and hence $F(s) =0$.
3. Cells have limited compressibility and push each other for centre–centre distances smaller than the rest length to minimize their overlap, i.e. $F(r) < 0$ for $r < s$.
4. Forces are either zero or cells exert a pull on each other due to cell–cell adhesion forces for distances larger than the rest length, i.e. $F(r) \ge 0$ for $r > s$.
5. There exists a maximum interaction range $r_A$ beyond which cells do not interact mechanically with one another, i.e. $F(r) = 0$ for $r \ge r_A$.

The simplest force function fulfilling these assumptions is a linear spring, used in early works by Drasdo and others (Drasdo 2000; Drasdo and Loeffler 2001). For the linear spring, the force is assumed to be directly proportional to the distance between cell midpoints, vanishing at the rest length. While this behaviour may be reasonable for small distances, it results in unphysically strong long-range interactions if extended to distances larger than the rest length. At the same time, a linear force does not result in very large repulsive forces when cells are very close, i.e. cells are highly compressible. This can lead to the problem of collapsing volumes, where a cell population collapses on itself under strong compression (Pathmanathan et al. 2009). Additionally, the linear force will feature a large discontinuity at the maximum interaction range which can lead to further numerical difficulties. For both these reasons, force functions are favoured which feature (i) stronger repulsive interactions for cell separation distances close to zero and (ii) adhesive interactions that vanish for large cell distances. Examples of force functions with these properties include classical potentials such as the Morse or the Lennard-Jones potential (Atkins et al. 2018), physically motivated force functions such as Hertz (Johnson 1985) and a number of mathematical functions built by extending the linear force with different terms. Table 1 summarizes the mathematical equations for several cell–cell interaction functions used for CBMs and examples of their references in literature.

Note that our assumptions detailed above do not include a hysteresis effect where the adhesion between cells depends on whether they were in contact before or not. Such an effect is modelled by the Johnson–Kendall–Roberts (JKR) model for the deformation of elastic bodies, which has been confirmed experimentally to be valid for biological cells under certain conditions (Chu et al. 2005). In addition to exhibiting a different biomechanical behaviour than what we consider in this study (as detailed in assumptions 1–5 above), the JKR force is also computationally more expensive than the other functions listed in Table 1. This is because the JKR force requires the solution of an implicit equation in order to recover the force for a given centre–centre distance (Byrne and Drasdo 2008). To decrease the computational requirements, the JKR can be approximated by a third-order polynomial as done in CellSys (Hoehme and Drasdo 2010b, c). It would generally be possible to include a kind of simplified hysteresis effect for the force functions we consider here, by applying an adhesive force only if cells are moving apart, but not when they are approaching each other. We leave this approach and its evaluation, along with a comparison to the JKR force, for future work.

In what follows we focus on the cubic, the piecewise polynomial and the generalized linear spring force. These pairwise interaction forces are those implemented as default forces in the open-source software packages MecaGen (Delile et al. 2017), PhysiCell (Ghaffarizadeh et al. 2018) and Chaste (Cooper et al. 2020), and therefore, it is likely that these functions are those that modellers new to the field come into contact with first. In the following subsections we describe the three chosen force functions and study if and how they can be parameterized based on pairwise interactions in order to produce similar biological behaviour at the population level. We will then use this parameterization as a starting point for a quantitative comparison of key numerical properties of CBM simulations using these force functions in Sect. 4.

**Table 1:** Overview over cell–cell interaction functions used for CBMs

Name	Mathematical definition	Parameters	Note/reference
Linear	$$F^{\text {lin}}(r) = {\left\{ \begin{array}{ll} \mu (r-s) &{} \text {if } r \le r_A,\\ 0 &{} \text {otherwise}.\end{array}\right. }$$	$\mu$: spring stiffness coefficient, $s$: rest length, $r_A$: maximum interaction distance	Used in (Drasdo 2000; Drasdo and Loeffler 2001).
Hertz	$$F^{\text {Hertz}}(r) = {\left\{ \begin{array}{ll} - \mu (s-r)^{\frac{3}{2}} &{} \text {if } r \le s,\\ 0 &{} \text {otherwise}. \\ \end{array}\right. }$$	$\mu$: spring stiffness coefficient, $s$: rest length	Repulsion only. Used in (Schaller and Meyer-Hermann 2005; Galle et al. 2005).
Johnson–Kendall–Roberts (JKR)	$F^{\text {JKR}}(a) = \left( \frac{4}{3} a^3 - \sqrt{8 a^3}\right) , \text {where } a^2 - \sqrt{2 a} = s-r$	$s$: rest length	Non-dimensionalized form. The second equation must be solved iteratively for the radius of the contact surface $a = a(r)$ for a given value of $r$. Used in (Byrne and Drasdo 2008; Hoehme and Drasdo 2010b).
Generalized linear spring	$$F^{\text {GLS}}(r) = {\left\{ \begin{array}{ll}\mu \log (1+(r-s)) &{} \text {if } r \le s,\\ \mu (r-s) \exp (-a (r-s)) &{} \text {if } s < r \le r_A,\\ 0 &{} \text {otherwise}.\end{array}\right. }$$	$\mu$: spring stiffness coefficient, $s$: rest length, $a$: controls the width, $r_A$: maximum interaction distance	Functional form of the interaction force used in Chaste (Cooper et al. 2020) (release 2019.1). Continuous but not differentiable at $r=s$. Discontinuous at the cut-off value $r_A$.
Cubic	$$F^{\text {cubic}}(r)= {\left\{ \begin{array}{ll} \mu \left( r - r_A\right) ^2 \left( r - s \right) &{} \text {if } r \le r_A, \\ 0 &{}\text {otherwise}. \end{array}\right. }$$	$\mu$: spring stiffness coefficient, $s$: rest length, $r_A$: maximum interaction distance	Functional form of the interaction force used in MecaGen (Delile et al. 2017). The quadratic term approximates the contact area.
Piecewise polynomial	$$F^{\text {poly}}(r) = {\left\{ \begin{array}{ll} \begin{aligned} \mu _{A} &{} \left( 1 - \dfrac{r}{r_A} \right) ^{n+1} \\ &{} - \mu _{R} \left( 1 - \dfrac{r}{r_R} \right) ^{p+1} \end{aligned} &{} \text {if }r\le r_R, \\ \mu _{A} \left( 1 - \dfrac{r}{r_A} \right) ^{n+1} &{} \text {if }r_R < r\le r_A,\\ 0 &{} \text {otherwise}.\\ \end{array}\right. }$$	$\mu _A$, $\mu _R$: spring stiffness coefficients, $r_R$: maximum repulsive interaction distance, $r_A$: maximum adhesive interaction distance	General functional form of the interaction force used in PhysiCell (Ghaffarizadeh et al. 2018). As a default, $n = p =2$. For $r_A$ fixed, $r_R$ and the ratio $\mu _A/\mu _R$ can be chosen s.t. $F(s)=0$.

These scalar functions are extended to 2D or 3D by multiplication with the normalized direction vector between cell midpoints, i.e. we define the force vector as ${\mathbf{F}}_{ij} = F(\Vert {\mathbf{r}}_{ij} \Vert ) \frac{{\mathbf{r}}_{ij}}{\Vert {\mathbf{r}}_{ij} \Vert }$, where ${\mathbf{r}}_{ij}={\mathbf{x}}_{j}-{\mathbf{x}}_{i}$. In this paper, we focus on comparing the GLS, cubic and piecewise polynomial forces

### Description of the Mathematical Form of the Force Functions and Their Parameters

In their respective software packages, these force functions are stated and parameterized very differently, so we start by describing each of them within a consistent notation and parameterization. In what follows, let $r$ denote the centre–centre separation distance between a pair of cells, $s$ the rest length and $r_A$ the maximum (adhesive) interaction distance. The maximum interaction distance $r_A$ and the rest length $s$ are parameters that relate to the biophysical properties of cells and are treated as fixed parameters given as initial data to a simulation.

The first of the three force functions we have chosen, the cubic force, combines a linear spring term with a quadratic term approximating the contact area of two overlapping spheres. It is used in the software MecaGen (Delile et al. 2017) and can be written as

10 $$\begin{aligned} F^{\text {cubic}}(r)= {\left\{ \begin{array}{ll} \mu \left( r - r_A\right) ^2 \left( r - s \right) &{} \text {if } r \le r_A, \\ 0 &{}\text {otherwise}. \end{array}\right. } \end{aligned}$$

**Fig. 3:**
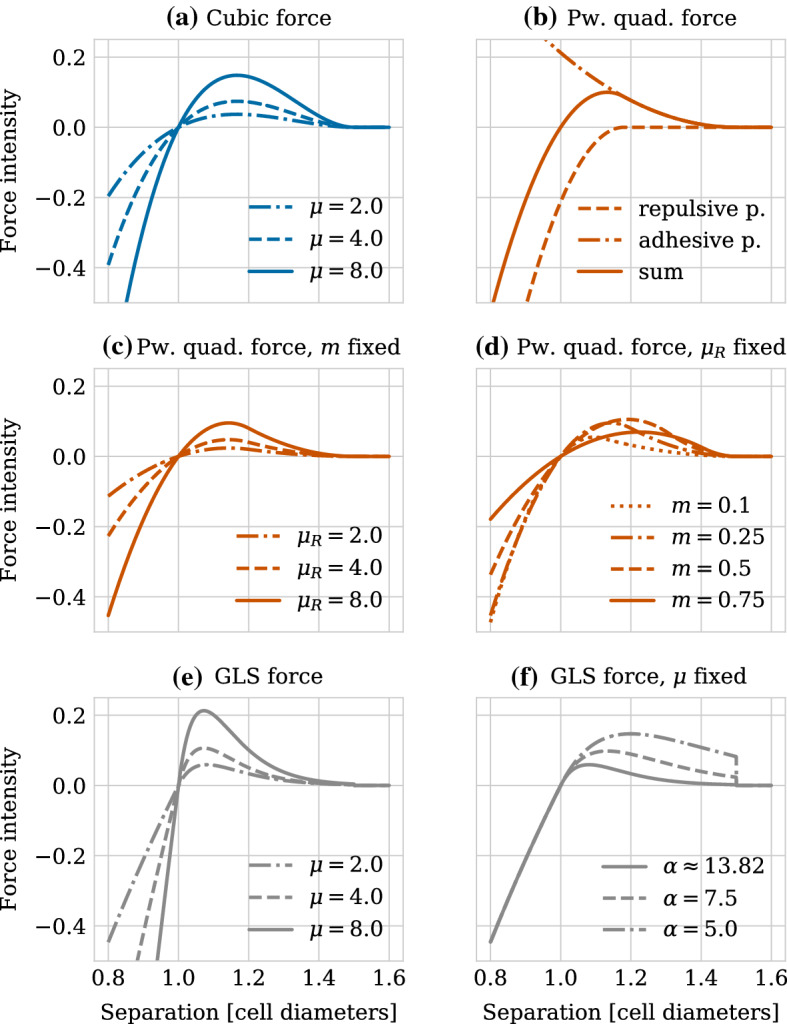
Intensity of the different forces as a function of cell separation distance. (**a**) Intensity of the cubic force shown for different spring stiffness values $\mu$. (**b**–**d**) Intensity of the piecewise quadratic force. (**b**) The repulsive and adhesive polynomials are shown individually. Parameters were chosen as $\mu _R=9.1$, $m=0.21$. (**c**) A fixed ratio $m=0.21$ was chosen and different spring stiffness values $\mu _R$. (**d**) A fixed spring stiffness value $\mu _R=8.0$ was chosen and different ratios *m*. In contrast to the cubic and GLS force, the piecewise quadratic force allows for varying the repulsive and adhesive spring stiffness parameter independently within a certain range. (**e**–**f**) Intensity of the GLS force with (**e**) different spring stiffness values $\mu$ and (**f**) $\mu =2.0$ and different values of the breadth $\alpha$. For the largest $\alpha$ here (solid line), as well as in (**e**), $\alpha$ is chosen according to Eq. (14)

Here, the only free parameter $\mu$ denotes the spring stiffness parameter. In order to ensure differentiability of the force at the rest length, i.e. for $r=s$, $\mu$ has to be independent of whether cells are overlapping ($r<s$) or not ($s\le r<r_A$). Larger values of $\mu$ increase the steepness of the repulsive interaction part, making cells behave more rigidly. At the same time, the magnitude of the adhesive interaction is increased, see Fig. 3a.

The second force function, the piecewise polynomial force is constructed as the sum of a positive adhesive and a negative repulsive polynomial, the support of which overlap, see Fig. 3b. As a default, quadratic polynomials are used in PhysiCell (Ghaffarizadeh et al. 2018). We take the same approach, leading to the formula,

11 $$\begin{aligned} F^{\text {pwq}}(r) = {\left\{ \begin{array}{ll} \mu _{A} \left( 1 - \dfrac{r}{r_A} \right) ^{2} - \mu _{R} \left( 1 - \dfrac{r}{r_R} \right) ^{2} &{} \text {if }r\le r_R, \\ \mu _{A} \left( 1 - \dfrac{r}{r_A} \right) ^{2} &{} \text {if }r_R < r\le r_A,\\ 0 &{} \text {otherwise}.\\ \end{array}\right. } \end{aligned}$$

Here, $$r_R$$ denotes the maximum repulsive interaction distance, and $$\mu _A$$ and $$\mu _R$$ are the adhesive and repulsive spring stiffness parameters. Note that in general $$ s< r_R < r_A$$ and $$\mu _A < \mu _R$$. As stated earlier, we require that $$F(s) = 0$$, i.e. that the force vanishes at the rest length. This effectively fixes the maximum repulsive interaction distance $$r_R$$ as a function of the ratio $$m= \frac{\mu _A}{\mu _R}$$ of the adhesive to the repulsive spring stiffness,

12 $$\begin{aligned} r_R = \frac{s}{1-\sqrt{m} (1-\frac{s}{r_A})}. \end{aligned}$$

Consequently, the piecewise quadratic function has two free parameters, $\mu _R$ and $m$. Figure 3c and d shows the force intensity as a function of cell separation distance for different parameter combinations. We observe that for a fixed ratio $m$, increasing the repulsive spring stiffness $\mu _R$ increases the magnitude of the force, similarly to the cubic force function (see Fig. 3c). On the other hand, Fig. 3d shows that the piecewise quadratic force function lets the modeller tune the level of adhesive interaction for a fixed repulsive strength within a certain range, which is not possible for neither the cubic nor the generalized linear spring.

Last but not least, the generalized linear spring (GLS) force as implemented in Chaste (Cooper et al. 2020) uses a logarithmic force for the repulsive regime. For cell distances larger than the rest length, it extends this force with a linear spring term multiplied with an exponential term to ensure decay of the force for large distances.

13 $$\begin{aligned} F^{\text {GLS}}(r) = {\left\{ \begin{array}{ll}\mu \log (1+(r-s)) &{} \text {if } r \le s,\\ \mu (r-s) \exp (-\alpha (r-s)) &{} \text {if } s < r \le r_A,\\ 0 &{} \text {otherwise}.\end{array}\right. } \end{aligned}$$

Contrary to the cubic and the piecewise quadratic force function, the GLS force is not constructed in a way that it vanishes for cell separation values larger than the maximum interaction distance. Instead, the force is just cut off at that value, leading to a discontinuity at $r=r_A$.

The GLS force has two free parameters, the spring stiffness $\mu$ and the parameter $\alpha$ which controls the width of the exponential decay in the adhesive regime. Similarly to the cubic function, ensuring differentiability forces the spring stiffness $\mu$ to be the same for the repulsive and the adhesive regime. Figure 3e and f shows the intensity of the GLS force for different values of $\mu$ and $\alpha$. We observe that as with the other force functions, increasing the spring stiffness $\mu$ leads to stronger repulsive and adhesive interactions (for a fixed value of $\alpha$) (see Fig. 3e). Decreasing $\alpha$ for a fixed spring stiffness value $\mu$ increases the amplitude of the adhesive interaction while not affecting the repulsive interaction (see Fig. 3f). However, it leads to a larger discontinuity at the maximum interaction distance $r_A$ which will affect the numerical properties of the GLS force as we will see in Sect. 4.3. It is possible to choose $\alpha$ dependent on $\mu$ in a way to ensure that the magnitude of the force is small at the maximum interaction distance $r_A$. Requiring e.g. that $F(r_A) \le \epsilon =10^{-3}$ leads to the following expression for $\alpha$,

14 $$\begin{aligned} \alpha = \frac{-1}{r_A-s} \log \left( \frac{\epsilon }{\mu (r_A-s)}\right) . \end{aligned}$$

Having described the force functions individually, we will now move on to compare their qualitative behaviour.

### Comparison of the Qualitative Behaviour for a Fixed Relaxation Time

The three studied force functions all have different functional forms and a varying number of free parameters. A natural question faced by a modeller is to what extent the force functions are interchangeable, i.e. whether they will result in simulations leading to the same biological conclusions given an appropriate parameterization. To study this, we first use a simple model system consisting of the fundamental unit of two interacting cells. We then check that slight differences at the level of pairwise interaction do not have a strong impact on a population level metric for two dimensional planar growth.

Since in this section we are interested in a comparison of the qualitative behaviour of the force functions and not (yet) their numerical properties, we solve the update equations for the cell positions by using the numerical solver provided by the solve_ivp function from the *scipy.integrate* library (Virtanen et al. 2020). As a default, this function provides an explicit high-level Runge–Kutta method of order 5(4) (Dormand and Prince 1980), which for the purposes of this section we assume to accurately resolve the cellular dynamics.

**Fig. 4:**
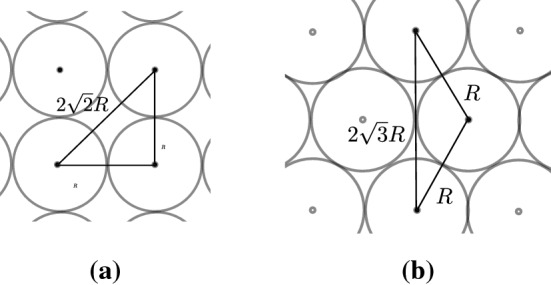
Illustration of cell centre–centre distances for populations arranged on (**a**) a Cartesian grid and (**b**) a hexagonal lattice in a so-called honeycomb configuration

In all experiments, we (i) let cells have a fixed radius of $R = 0.5$ cell diameters and (ii) fixed the rest length $s$ and the maximum interaction distance $r_A$ to the same value for all force functions. We chose the rest length to equal one cell diameter, i.e. $s = 2R = 1.0$ cell diameter. To ensure that a cell configuration placed at Cartesian coordinates relaxes to a honeycomb-like configuration, but that next-to-nearest neighbours do not interact at rest, the maximum interaction radius has to be chosen between $2\sqrt{2}R \approx 1.4$ and $2\sqrt{3}R\approx 1.7$ cell diameters, as illustrated in Fig. 4. We used $r_A = s + R = 1.5$ cell diameters.

To select the remaining free parameters, we assumed that all force functions should result in the same pairwise relaxation time after cell division. This can be seen as a basic requirement of interchangeable force functions for a proliferating system. However, it leads to differences in the transient relaxation dynamics in the repulsive regime, and also to differences in force magnitudes in the adhesive regime. First, we illustrate these differences. Then, we study their impact on a population level metric for a system of proliferating cells, the population radius and examine whether the fit can be improved by changes in the parameterization.

#### Pairwise Relaxation Dynamics

First, we considered two overlapping cells as they relax to mechanical equilibrium right after cell division. The cells were placed at a fixed separation distance $r_0= 0.3$ cell diameters and the system was evolved until the overlap was eliminated due to the repulsive forces (see Fig. 2 for an illustration). Parameters were chosen to ensure that for all force functions the relaxation time $t_0$ is equal to 1 h. Here, we defined the relaxation time as the time such that the distance between cells is equal to $99\%$ of the rest length $s$. The parameter values were determined numerically using the minimize function of the *scipy.optimize* library in combination with our CBMOS simulation code to calculate the separation distance as a function of the spring stiffness $\mu$. The resulting parameter values are stated in Table 2. Note that choosing a fixed relaxation time in this way does not determine the parameter $\alpha$ of the GLS force, as $\alpha$ only governs adhesive interactions. For the same reason, the choice of $\alpha$ will not affect the relaxation dynamics. We therefore postpone the discussion on how to choose $\alpha$ until later in this section when we consider how cells interact in the adhesive regime.

**Fig. 5:**
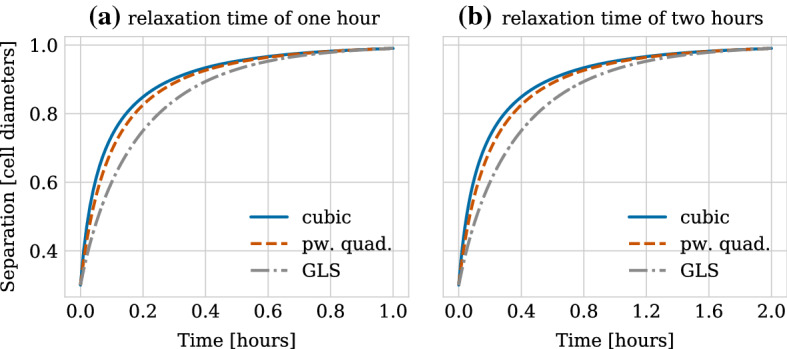
Relaxation dynamics between two cells initially placed at a fixed separation of 0.3 cell diameters. The parameters have been chosen such that the separation equals 0.99 cell diameters after (**a**) 1 h and (**b**) 2 h

Figure 5a shows the separation in cell diameters as a function of time for the different force functions. Over time the separation between the cells correctly approaches the rest length for all force functions. The rate at which the overlap is eliminated, however, differs between force functions. It decreases fastest for the cubic function and slowest for the GLS force. This means that cells behave more or less rigidly depending on the force function, even if they eliminate their overlap completely within the same time duration (same relaxation time). We will see in Sect. 4.1 that these differences in the stiffness of the system are reflected in the numerical stability bounds.

Doubling the relaxation time by halving the spring stiffness $\mu$ does not change the qualitative dynamics but scales the time scale, compare Fig. 5a to b. This can be seen analytically from stating the simple ODE for the distance between a pair of mechanically relaxing cells after division,

15 $$\begin{aligned} \dot{r} = -2 \mu \hat{F}(r), \end{aligned}$$

where we have made the dependence on the spring stiffness $\mu$ explicit by defining $\hat{F}(r) = \frac{1}{\mu }F(r)$. Rescaling time as $\tau = \mu t$ leads to $\dot{r}(\frac{t}{\mu }) = -2 \hat{F}(r(\frac{t}{\mu }))$, meaning that increasing $\mu$ speeds up the dynamics and decreases the relaxation time. Consequently, a longer relaxation time decreases the stiffness of the system at the cost of having to run simulations for a longer time. In the following, we considered the time scale on which mechanical relaxation happens to be (arbitrarily) fixed as 1 h.

**Table 2:** Parameter values for a fixed relaxation time $t_0$ of 1 h after cell division

Parameter	Description	Value
$\varvec{\mu _\mathbf{cubic }}$	Spring stiffness, cubic force	5.7
$\varvec{\mu _R}$	Repulsive spring stiffness, pw. quad. force	9.1
$\varvec{m}$	Ratio of adhesive to repulsive spring stiffness, pw. quad. force	0.21
$\mu _A$	Adhesive spring stiffness, pw. quad. force	1.91
$r_R$	Repulsive interaction distance, pw. quad. force	1.18
$\varvec{\mu _\mathbf{GLS }}$	Spring stiffness, GLS force	1.95
$\varvec{\alpha ^{(1)}}$	Breadth of exponential, GLS force (strategy #1)	7.51
$\varvec{\alpha ^{(2)}}$	Breadth of exponential, GLS force (strategy #2)	13.76

Free parameters are printed in bold. The spring stiffness values $\mu _{\text {cubic}}$, $\mu _R$ and $\mu _{\text {GLS}}$ and the ratio $m$ have been determined numerically by minimizing the difference between the separation distance *r* and $99\%$ of the rest length *s* at $t_0$, i.e. $\min _\mu \Vert r(t_0; \mu ) - 0.99 s \Vert$. Here, the minimization was done using the minimize function of the *scipy.optimize* library (Virtanen et al. 2020; The SciPy Community 2018), where the separation distance was evaluated for different spring stiffness values $\mu$ using our CBMOS simulation code. For the cubic and the GLS forces the BFGS algorithm (Nocedal and Wright 2006) and the Nelder–Mead algorithm (Nelder and Mead 1965; Wright 1996) were used, respectively. For the piecewise quadratic force, the minimization was done jointly over $\mu _R$ and the ratio $m$ using the L-BFGS-B algorithm (Byrd et al. 1995; Zhu et al. 1997). Values were rounded to two decimal values, except for the ratio where they were rounded to three decimal values. The truncated values were used in the calculation of the remaining parameters and all subsequent numerical experiments. The adhesive spring stiffness was chosen as $\mu _A = m \cdot \mu _R$. The value for $r_R$ was chosen according to Eq. (12). The value for $\alpha ^{(1)}$ was determined by fitting the GLS force to the cubic force in magnitude over the adhesive regime with all other parameters fixed as listed in the table (strategy #1). The alternative value, $\alpha ^{(2)}$, was chosen according to Eq. (14)(strategy #2). The values for $r_R$ and $\alpha ^{(2)}$ are stated here rounded; however, the exact values were used in the numerical experiments

Next, we asked how cells interact in the adhesive regime when parameterized to have the same fixed relaxation time. As explained above, this parameterization does not determine the $\alpha$ parameter responsible for tuning the breadth of the adhesive interactions of the GLS force. Instead, we compared two strategies for choosing $\alpha$. Firstly, we fit the GLS force to the cubic force in magnitude over the adhesive regime (strategy #1). Here, our motivation was to make the force functions as qualitatively similar for adhesive interactions as possible while still ensuring the same fixed relaxation time. We chose to fit to the cubic force rather than the piecewise quadratic force due to the simpler mathematical form and smaller number of free parameters of the former. Since $\alpha$ only affects adhesive interactions, fitting over the complete regime would have resulted in the same parameter value. We list this $\alpha$ in Table 2 as $\alpha ^{(1)}$. Secondly, we chose $\alpha$ according to Eq. (14) to ensure that the discontinuity of the GLS force at the maximum interaction radius is small (strategy #2). We list this $\alpha$ in Table 2 as $\alpha ^{(2)}$.

**Fig. 6:**
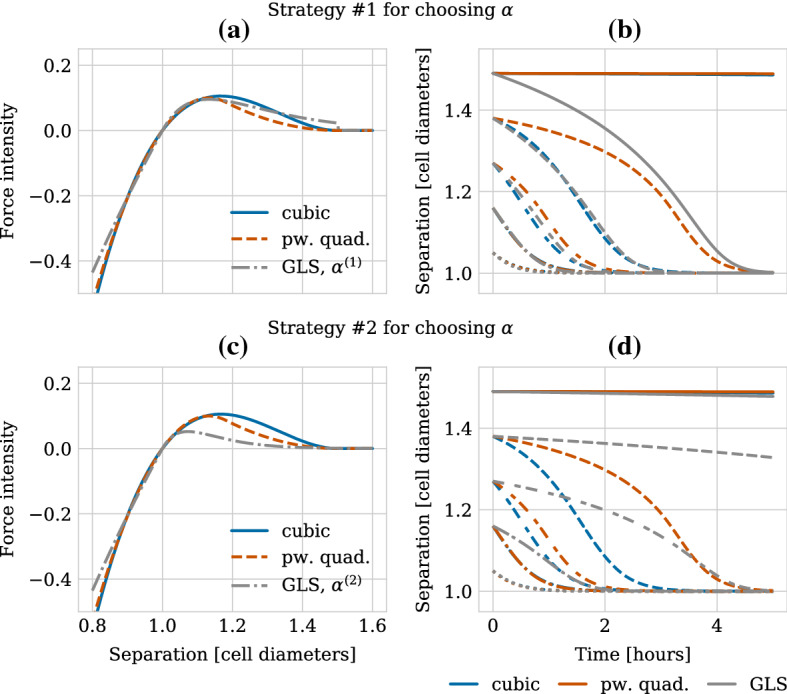
(**a**) Intensity of the different force functions as a function of cell separation distance for the adhesive regime. (**b**) Relaxation dynamics between adhering cells under the different force functions. Two cells are initially placed at different distances within the maximum interaction distance of 1.5 cell diameters. Different linestyles correspond to different initial separation distances between the two cells. Parameters for both (**a**) and (**b**) have been chosen such that the force functions have the same relaxation time of 1 h after cell division as listed in Table 2

Figure 6 shows the intensity of the force functions as a function of cell separation (left column) and the resulting dynamics of adhering cells placed initially at different centre–centre distances larger than the rest length (right column). The results for choosing $\alpha$ according to strategy #1 are shown in the upper row and those for strategy #2 in the lower row. Note that the force intensities and the separation dynamics shown for the cubic and the piecewise quadratic forces are the same in both rows, as is the force intensity for the GLS force over the repulsive regime, i.e. for $r<s$. We can therefore observe in both Fig. 6a and c that the cubic and the piecewise quadratic force agree well in the repulsive regime, whereas the GLS force agrees less well for separation values of $r<0.85$ cell diameters. Furthermore, the cubic and the piecewise quadratic forces have a similar maximum intensity in the adhesive regime, i.e. for $r>s$, which is attained for slightly smaller cell separation distances for the piecewise quadrative force than for the cubic force. The piecewise quadratic force then shows a steeper decrease to zero magnitude at the maximum interaction distance. This leads to the transient dynamics of adhering cells agreeing well between the cubic and the piecewise quadratic force for cells placed at separation distances either close to the maximum interaction distance or closer than roughly 1.3 cell diameters (Fig. 6b, d). For intermediate separation distances, cells adhere faster when simulated with the cubic force.

We now turn to the GLS force. Let us first consider strategy #1 for choosing $\alpha$. In Fig. 6a, we observe that while all three force functions are similar in maximum amplitude in the adhesive regime, the separation values for which the maximum is attained agree better between the GLS and the piecewise quadratic forces. On the other hand, the cubic and the GLS forces start off with a similar decrease; however, the GLS force does not decrease to zero at the maximum interaction distance, but is discontinuous with a jump of about 0.02 (for the given parameterization). For separation values close to the rest length, the transient dynamics of adhering cells for the GLS force agree well with those for the other two force functions (Fig. 6b). For intermediate separation values, the transient dynamics generated with the GLS force agree well with those generated using the cubic force. However, we observe that due to the differences in force magnitude and slope close to the maximum interaction value, the transient dynamics differ qualitatively for the three force functions for cells placed just within interaction distance of one another. More specifically, for cells that initially were placed just 0.01 cell diameters from the maximum interaction distance, the use of the GLS force results in these cells touching each other after 5 h. The dynamics of those cells simulated with the cubic and the piecewise quadratic forces, on the other hand, remain close to the maximum interaction distance for even longer time scales.

We can recover the robustness of the transient dynamics to small perturbations of the maximum interaction value for the GLS force by changing the parameterization strategy for $\alpha$ to strategy #2 as seen in Fig. 6d. In this case, the force intensity is small at the maximum interaction radius (Fig. 6c). However, this leads to the GLS force having a much lower maximum intensity in the adhesive regime which is attained also for much smaller separation values than the maxima for the cubic and the piecewise quadratic forces. As a result, the transient dynamics for adhering cells differ qualitatively between the GLS force and the other two forces over a large range of separation values.

In the next section, we will turn to investigate how these qualitative differences between the forces—and in particular between the parameterization strategies for $\alpha$ for separation values close to the maximum interaction value—affects a freely growing monolayer under compression from high proliferation, where the repulsive interactions play the major role, but adhesion between next-to-nearest neighbours is also present.

#### Impact of the Parameterization on the Radius of a Monolayer Population Relaxing After Intense Proliferation

As discussed in the previous subsection, calibrating the force functions to agree on the mechanical relaxation time is a simple and computationally cheap procedure. But since the parameterization is based on a summary statistic, the forces disagree quantitatively both in the strongly compressed repulsive regime and in the adhesive regime. The timescale for which the forces show large disagreement in the repulsive regime is relatively short compared to the relaxation time. As can be seen in Fig. 6a and c the force functions agree well in the repulsive regime at a separation of 0.85 cell diameters or more. In Fig. 5, we see that this separation is reached in less than the first $30\%$ of the total relaxation time for both the shorter and longer relaxation times. The disagreement in the adhesive regime is larger, especially when $\alpha$ is chosen according to strategy #2. In this section, we investigate how these disagreements affect the relaxation of a small growing monolayer. As we are interested in how the force functions compare when modelling mechanical relaxation due to proliferation of the tissue, we choose a setting of high compression by letting all cells divide simultaneously.

**Fig. 7:**
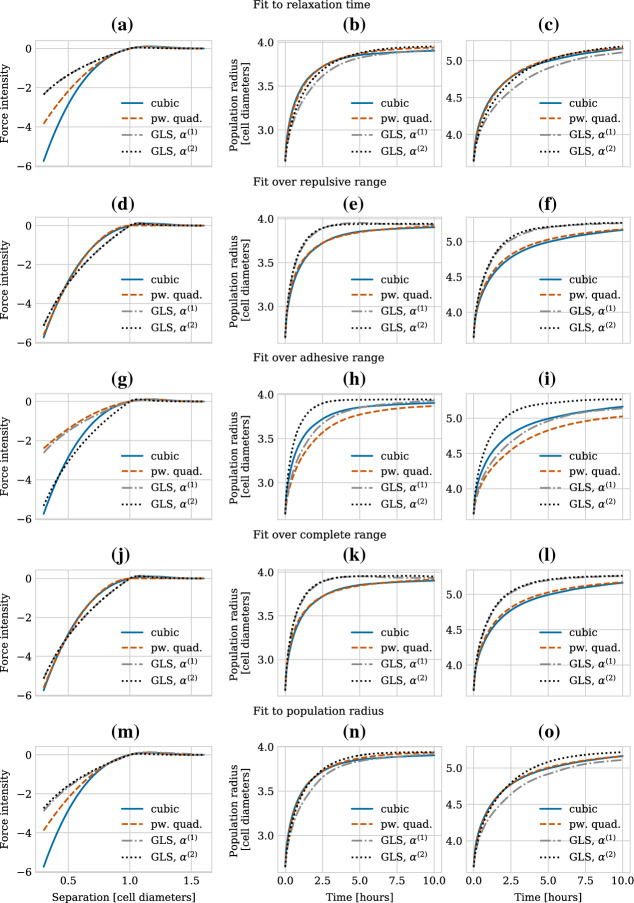
Plot showing force intensities for four parameterization strategies (left column) and corresponding simulation results for the population level radius of a growing monolayer in two dimensions of initially 19 cells (middle column) and of initially 37 cells (right column). In both settings each cell divided at the beginning of the simulation, doubling the number of cells to 38 cells (middle column) and 74 cells (right column), respectively. The population radius for each time point was calculated as $r = R + \max _i \Vert \mathbf{x} _i - \frac{1}{K} \sum _{i=1}^{K} \mathbf{x} _i \Vert _2$, where $R=0.5$ cell diameters is the radius of a single cell and *K* denotes the total number of cells. The population radius was then averaged over 10 simulation runs with different random seeds. (**a**)–(**c**) Fit to relaxation time, (**d**)–(**f**) fit over repulsive range, (**g**)–(**i**) fit over adhesive range, (**j**)–(**l**) fit over complete range, (**m**)–(**n**) fit to population radius for 38 cells, (**o**) population radius for 74 cells using the parameter values fit to a population radius for 38 cells as shown in (**m**) and (**n**). The individual parameter values used can be found in Tables 2 and 3

**Fig. 8:**
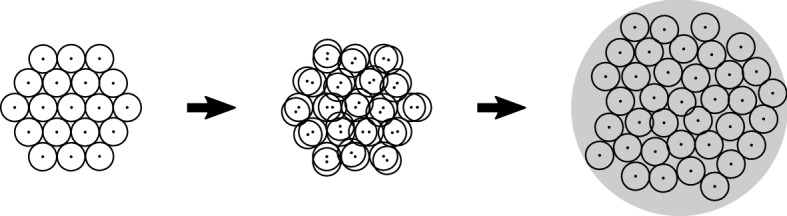
Illustration of a monolayer growing and relaxing under intense proliferation. Initially, 19 cells are placed in a honeycomb arrangement (left). Then, all cells are simultaneously let to divide (middle). The resulting population of 38 cells is mechanically relaxed to equilibrium and the population radius tracked over the process (right). Note that due to adhesive forces present the equilibrium state can include slight overlaps between cells. The procedure is done similarly for an initial placement of 37 cells (not shown)

In Fig. 7, we show force intensities for five (main) parameterization strategies (left column) and corresponding simulation results for the population level radius of a growing monolayer in two dimensions (middle and right columns). In each panel, we show data for both (sub-)parameterization strategies for choosing the $\alpha$ parameter for the GLS force (denoted by “GLS, $\alpha ^{(1)}$” and “GLS, $\alpha ^{(2)}$”). In the middle column, we initialised 19 cells arranged in a honeycomb pattern. We then let all cells divide simultaneously at the beginning of the simulation and tracked the population radius over 10 h (in-simulation time). During this time, the resulting monolayer of 38 cells relaxed to a mechanical equilibrium configuration. Figure 8 shows an illustration of the procedure. For the right column, we did the same procedure for an initial honeycomb pattern of 37 cells, resulting in a final population size of 74 cells, approximately doubling the number of cells in our first experiment. This was done in order to empirically check the robustness of our results to the size of the growing monolayer. In both cases, the population radius was calculated by taking the maximum distance of any cell to the centroid of the population and adding the radius $R$ of a single cell. The exact parameter values used for the different parameterization strategies are listed in Table 3. Note that fitting over the complete regime or only over the repulsive regime results in nearly identical parameters (see discussion below).

First of all, we note that our experiments showed that independently of the exact parameterization used, all three forces are robust to the issue of collapsing volumes discussed in Pathmanathan et al. (2009), since in no case the population radius decreases to values smaller than that of the initial configuration (see all subplots of Fig. 7).

Secondly, we observe that matching relaxation times after cell division results in good agreement for all force functions on the population level metric despite large force discrepancies present in the highly compressed regime, as can be seen in Fig. 7a, b and c. Empirically, this seems to hold true independently of the actual size of the monolayer, as the curves agree very well for both monolayer sizes of initially 19 and 37 cells. Interestingly, the agreement for the GLS force is better when using strategy #2 for choosing $\alpha$ (discontinuity in GLS force small at maximum interaction value) than when using strategy #1 (forces agree well in intensity over the adhesive regime).

Fitting the force functions by minimizing the discrepancy in force intensity over the repulsive regime works well for the cubic and the piecewise quadratic forces but results in larger differences in population radius for the GLS force due to large force discrepancies in the medium-to-low compressed regime (see Fig. 7d and e). This holds true independently of which sub-parameterization strategy we choose for $\alpha$. The (main) parameterization strategy of fitting the forces in intensity over the adhesive regime again results in worse agreement (Fig. 7g and h). Unsurprisingly, the agreement between the GLS force and the cubic and piecewise quadratic forces is better when using strategy #1 for $\alpha$, than when using strategy #2. Nevertheless, the agreement is worse than for other main parameterization strategies. We also attempted to fit over the complete regime, i.e. both repulsive and adhesive, (Fig. 7j and k) but saw no improvement compared to fitting only over the repulsive regime. This can be explained by the fact that in our experimental set-up, where the monolayer relaxes after intense proliferation, most interactions will be of the repulsive kind.

Finally, we parameterized the functions by fitting to the actual population radius for the monolayer of initially 19 cells, Fig. 7m and n. The strategy here was to numerically fit the population radius obtained from simulations using the piecewise quadratic and GLS forces to the population radius averaged over 10 simulations with the cubic force. Interestingly, this approach resulted in similar force parameters to those obtained by matching relaxation times, compare Tables 2 and 3. Again, the agreement is better when using strategy #2 for choosing $\alpha$ than when using strategy #1. The increase in computational time needed to do the same numerical optimization procedure for the experimental set-up of initially 37 cells prohibited us from doing so. Instead we checked how the parameters from the smaller monolayer transferred to the larger monolayer in Fig. 7o. In contrast to when fitting to the relaxation time, the population radius does not agree well between the different forces for this case.

In conclusion, for the initial stages of two-dimensional monolayer growth, the three force functions we consider can all result in close macroscopic readouts (in this case the population radius) for translations of parameters such as repulsive spring stiffness. In this sense, they can be seen as interchangeable from a modelling perspective, at least for the model system we consider where repulsive interactions dominate over adhesive ones. Interestingly, fitting to the relaxation time leads to better agreement than when transferring parameters obtained from fitting directly to the population radius from a smaller to a larger population. In the case of the GLS force, we obtained this good agreement when choosing the $\alpha$ parameter such that the discontinuity was small at the maximum interaction value (strategy #2). This indicates that for model systems under high compression where repulsive interactions play the major role, robustness to small perturbations in the maximum interaction radius is more important than good agreement between force intensities in the adhesive regime. Therefore, we chose to continue using strategy #2 in all subsequent numerical experiments, unless explicitly stated otherwise.

Having established a way of effectively parametrizing the three forces using the simple pairwise metric of relaxation time, we now turn to discuss how the forces compare numerically. We will see that the fact that the force intensities differ in the highly compressed regime can have an impact on the numerical solution of the equations of motion.

**Table 3:** Parameter values for different fitting procedures

Fitting procedure	$\varvec{\mu _R}$	$\varvec{m}$	$\mu _A$	$r_R$	$\varvec{\mu _\mathbf{GLS }^{(1)}}$	$\varvec{\alpha ^{(1)}}$	$\varvec{\mu _\mathbf{GLS }^{(2)}}$	$\varvec{\alpha ^{(2)}}$
Fit over complete range	11.33	0.002	0.023	1.015	4.27	11.41	4.27	15.33
Fit over repulsive range	11.35	0.002	0.023	1.015	4.27	11.41*	4.27	15.33
Fit over adhesive range	8.13	0.462	3.740	1.292	2.18	7.95	4.43	15.41
Fit to population radius of initially 19 cells	9.76	0.260	2.538	1.205	2.40	6.64	2.28	14.08

Free parameters are printed in bold. The spring stiffness values $\mu _R$ and $\mu _{GLS}$, the ratio $m$ and the breadth $\alpha$ were determined numerically by minimizing the difference between force function magnitudes for different ranges of cell separation values $r$. For the fit over the repulsive range we chose $r= [0.3, 1.0]$, for the fit over the adhesive range $r= [1.0, 1.5]$ and for the fit over the complete range $r= [0.3, 1.5]$. In all cases, the cubic force with parameters as stated in Table 2 was used as a reference. The minimization was done using the minimize function of the *scipy.optimize* library (Virtanen et al. 2020; The SciPy Community 2018). Values were rounded to two decimal values, except for the ratio where they were rounded to three decimal values. The truncated values were used in the calculation of the remaining parameters and all subsequent numerical experiments. For the piecewise quadratic force, the minimization was done jointly over $\mu _R$ and the ratio $m$. The adhesive spring stiffness was chosen as $\mu _A = m \cdot \mu _R$. The value for $r_R$ was chosen according to Eq. (12). Its value is stated here rounded to three decimals; however, the exact value was used in the numerical experiments. For the GLS force, the optimization was done jointly over $\mu _\text {GLS}$ and $\alpha$ when using strategy #1 (values denoted by $\mu _\text {GLS}^{(1)}$ and $\alpha ^{(1)}$), except for the case of fitting over the repulsive range (the value in question is denoted by a * in the table). There, only the value of $\mu _{\text {GLS}}^{(1)}$ was optimized for in a first step, since $\alpha$ does not affect repulsive interactions. Then using that fixed spring stiffness value, $\alpha ^{(1)}$ was determined by fitting over the adhesive regime. Note that this results in identical parameter values as when fitting jointly directly over the complete regime. When using strategy #2 for $\alpha$ the optimization was done only over $\mu _\text {GLS}$ and $\alpha$ was then chosen according to Eq. (14) (values denoted by $\mu _\text {GLS}^{(2)}$ and $\alpha ^{(2)}$). For the fit to the population radius, the population dynamics were simulated for a given spring stiffness value using our CBMOS simulation code. Again, the dynamics obtained using the cubic force fitted to a relaxation time of 1 h were used as reference

## Numerical Properties of Three Popular Force Functions

Given a parameterization of the various force functions that results in similar population level behaviour, it is interesting to study the properties of the numerical solution of the corresponding ODE (6). In particular, we are interested in differences in the error as the system approaches equilibrium for given time step sizes $\varDelta t$, since this directly affects the simulation efficiency for any implementation of a CBM simulator. The large force gradients right after cell divisions (when cells might overlap to a large extent) causes the ODE system to be stiff. Commonly used explicit schemes such as the forward Euler method are simple to implement and avoid the expensive solution of large systems of nonlinear equations needed for implicit methods. However, explicit methods struggle with stiff systems because the time step will be forced to take small values in order to ensure stability and accurate resolution of the transient solution. We are primarily concerned with differences in the numerical properties depending on the force function chosen.

Similarly to the structure of Sect. 3, we start by examining the numerical stability for the case of pairwise relaxation dynamics in Sect. 4.1 and then relate this to the population level behaviour in Sect. 4.2. Motivated by our findings that ensuring only stability is insufficient, we then perform a convergence study in Sect. 4.3 where we compare first and second-order solvers.

### Requiring Numerical Stability is Not Enough to Prevent Unphysical Trajectories After Cell Division

The forward Euler method is the numerical scheme used most often in CBM implementations. Therefore, we started by studying the effect of the time step on the pairwise relaxation dynamics of cells after division, when simulated with this method. We followed the same experimental set-up as in Sect. 3.2.1, but now used our own implementation of the forward Euler method to numerically solve the update equation for the cell positions, as given in Eq. (7). Here, we were free to vary the time step $\varDelta t$ as necessary.

**Fig. 9:**
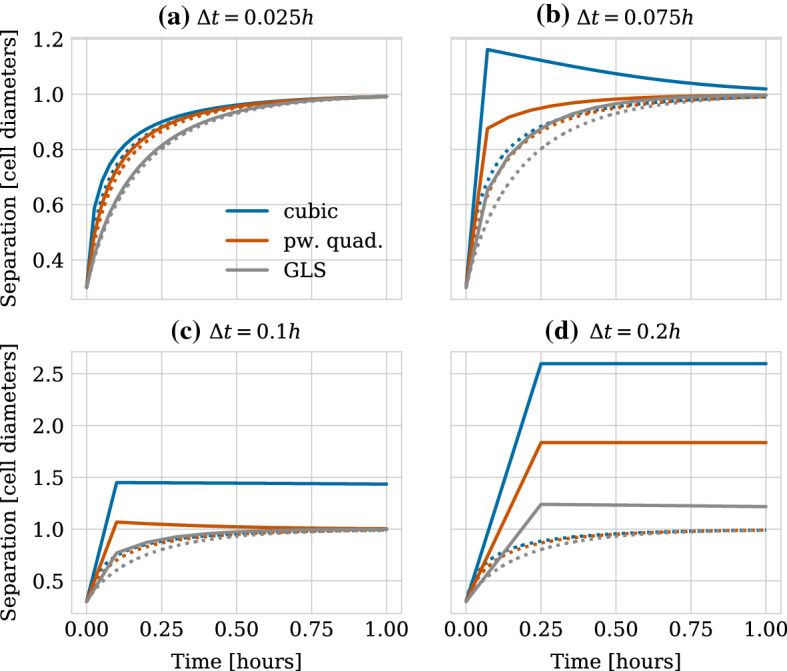
Stability of the numerical solution for the relaxation dynamics between daughter cells under different force functions. (**a**) $\varDelta t =0.025 h$, (**b**) $\varDelta t =0.075 h$, (**c**) $\varDelta t =0.1 h$, (**d**) $\varDelta t =0.2 h$. Note that the legend shown in panel (**a**) is valid for all panels. For reference, the dotted curves correspond to an accurate solution (less than 1% relative error) calculated using $\varDelta t = 0.005h$

We show the relaxation dynamics for the three force functions and different time steps in Fig. 9. We observe three types of behaviours of the numerical solution depending on the time step size. Firstly, if the time step is too large, the movement of the cells centres in one time step can be so large so that cells immediately separate and remain far from each other, independent of the duration of the simulation. This behaviour can be seen in Fig. 9d for the cubic and the piecewise quadratic forces. The reason is that the centre–centre distance exceeds the adhesion threshold. Secondly, for smaller time steps, we can observe dynamics where the cells overshoot but adhere and we eventually recover the correct separation distance at equilibrium (Fig. 9b for the cubic force, Fig. 9c for the cubic and piecewise quadratic forces and Fig. 9d for the GLS force). Numerically, this corresponds to a stable solution for a time step $\varDelta t$ smaller than a threshold $\varDelta t^*_\text {stab}$. However, this behaviour leads to unphysical cell trajectories after division. Therefore, it is important that the time step is small enough to ensure monotone, smooth and accurate relaxation of daughter cells following the division of the mother cell. This third behaviour can be seen in Fig. 9a for all three force functions, in Fig. 9b for the piecewise quadratic and the GLS force and in Fig. 9c only for the GLS force. From a numerical viewpoint, this desired behaviour ensuring physically realistic cell trajectories can only be observed for time steps below a certain threshold $\varDelta t^*_\text {mono}$. The exact thresholds for this critical time step are force function (and solver) dependent.

For the case of pairwise relaxation after cell division using the forward Euler method, as we consider here, the thresholds for monotonicity and stability can be calculated as

16 $$\begin{aligned} \varDelta t^*_{\text {mono}} = \frac{r_0-s}{2F(r_0)} \text { and } \varDelta t^*_{\text {stab}} = \frac{r_0-s}{F(r_0)}. \end{aligned}$$

Note that the monotonicity threshold is exactly half the stability threshold. These bounds can be derived as follows. Applying the forward Euler method, Eq. (7), to the update equation for the case of pairwise relaxation, Eq. (15), the expression for the iterates $r_n$ reads,

17 $$\begin{aligned} r_{n+1} = r_n - 2 \varDelta t F(r_n). \end{aligned}$$

To ensure stability, we require that the difference between the rest length $s$ and the cell separation distance $r$ is decreasing in absolute value for subsequent iterates, i.e. that

18 $$\begin{aligned} |s- r_{n+1} | \le |s - r_n|, \end{aligned}$$

(see also e.g. (Ascher and Petzold 1998) for a standard derivation of the (absolute) stability restrictions on the forward Euler method). The monotonicity bound is obtained by further requiring that the difference between the rest length $s$ and the cell separation distance $r$ is positive for all iterates meaning that the iterates $r_n$ do not overshoot, i.e.

19 $$\begin{aligned} 0 \le s - r_{n}. \end{aligned}$$

Since in our case the pairwise interaction force $F$ is negative and monotonically increasing for separation distances smaller than the rest length, i.e. for $r<s$, the bound will be determined by the restriction on $\varDelta t$ in the first step and hence will depend on the initial separation distance $r_0$ between cells after division. This means that we obtain Eq. (16) by evaluating Eq. (17) for $n=0$ and plugging it into Eqs. (19) and (18).

$\varDelta t^*_\text {mono}$ implies an upper limit on the error in the transient phase for pairwise relaxing cells in order to ensure physical division trajectories. When comparing different numerical schemes, this limit will be met for different $\varDelta t^*_\text {mono}$. The error, and hence $\varDelta t^*_\text {mono}$, depends both on the relaxation time (stiffness) and the local truncation error of the scheme. The force function chosen directly affects the stiffness of the system. Table 4 lists the threshold values for the three force functions. When fitted to have the same relaxation time, the GLS force allows for larger time step sizes than the piecewise quadratic force. The cubic force function has the smallest, i.e. the most restrictive bounds.

**Fig. 10:**
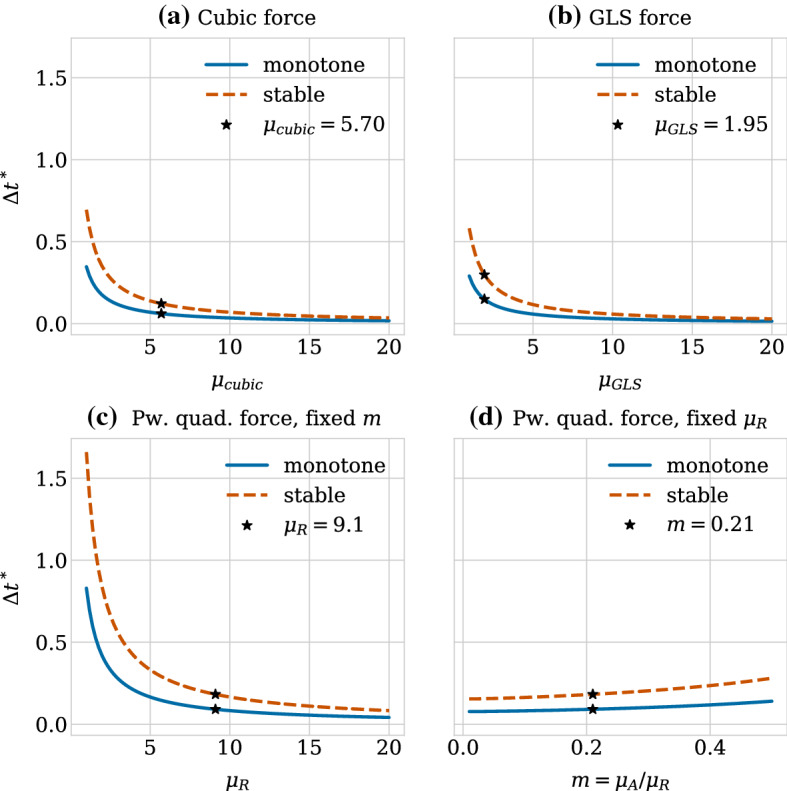
Stability and monotonicity bounds on $$\varDelta t$$ given by Eq. (16) as a function of force function parameters. The stars highlight the parameter values for which the force functions agree on a relaxation time of 1 h. (**a**) Cubic force, (**b**) GLS force, (**c**) piecewise quadratic force, fixed ratio $$ m = 0.21$$, (**d**) piecewise quadratic force, fixed repulsive spring stiffness $$\mu _R=9.1$$

In Fig. 10, we studied the dependence of the stability and monotonicity bounds on the free parameters of the three force functions. We observed that the qualitative dependence of the bounds on the spring stiffness parameter agrees across all three forces. However, the parameter point corresponding to a consistent relaxation time of 1 h is situated very differently on the curves, resulting in large quantitative differences in the threshold values for our parameterization.

**Fig. 11:**
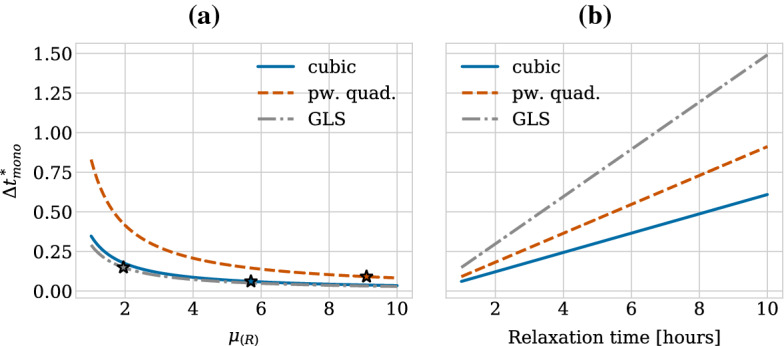
Comparison of the monotonicity bound $\varDelta t^*_{\text {mono}}$ (c.f. Eq. (16)) as a function of (**a**) the spring stiffness $\mu$, respectively, $\mu _R$ and (**b**) the relaxation time for the different forces. The stars highlight the parameter values for which the forces agree on a relaxation time of 1 h as given in Table 2 (using $\alpha ^{(2)}$). For the piecewise quadratic force, the ratio was fixed as $m = 0.21$

**Table 4:** Table containing the monotonicity bounds $\varDelta t^*_{\text {mono}}$ and stability bounds $\varDelta t^*_{\text {stab}}$ for the relaxation dynamics of pairwise overlapping cells when solved using the forward Euler method in combination with the different force functions

Force function	$\varDelta t^*_{\text {mono}}$	$\varDelta t^*_{\text {stab}}$
Cubic	0.0609	0.1218
Pw. quad.	0.0912	0.1823
GLS	0.1491	0.2982

The values were obtained by evaluating Eq. (16) for $r_0=0.3$ cell diameters and the force parameters according to Table 2 (using $\alpha ^{(2)}$) and are given in hours

This points to another related, important practical consideration for a modeller, namely that the numerical properties for any scheme will depend on the physical parameters of the force function. When conducting e.g. parameter sweeps to study the sensitivity of some metric of interest, it is important to remember this since if the same time step is used and it is too large, one can easily end up in situations where one starts violating the monotonicity condition. Figure 11a quantifies this by plotting the monotonicity threshold $\varDelta t^*_\text {mono}$ as a function of the spring stiffness parameter for the three force functions in a single plot. As can be seen, all force functions show the same qualitative behaviour as $\mu$ is varied. Since the spring stiffness $\mu$ scales inversely with the relaxation time $t_0$ (compare with the discussion in Sect. 3.2.1), the dependence of $\varDelta t^*_\text {mono}$ on the relaxation time is linear, as shown in Fig. 11b.

We can conclude that requiring stability of the numerical solution is insufficient for ensuring that cell trajectories are physically correct after division. Furthermore, we observed that the three forces put different restrictions on the time step size, with the GLS force allowing for the largest step sizes while still qualitatively correctly resolving the dynamics for daughter cells after division. We now move on to relate our findings for this case of pairwise interaction to the population level behaviour of a monolayer population under intense proliferation.

### Too Large Step Sizes can Lead to Geometrical Differences at the Population Level

In the previous section, we studied the case of pairwise interactions between cells. These bounds will not hold exactly for more complex systems because the underlying ODE system to be solved is more involved. Similarly to Sect. 3, we here empirically relate the findings for the pairwise case to the case of simulating the positions of cells and the population radius of a monolayer population. Although it is unclear how to precisely relate the two cases mathematically, one can expect that not ensuring physicality of the trajectories of the daughter cells after cell division will have an impact on the population level behaviour.

**Fig. 12:**
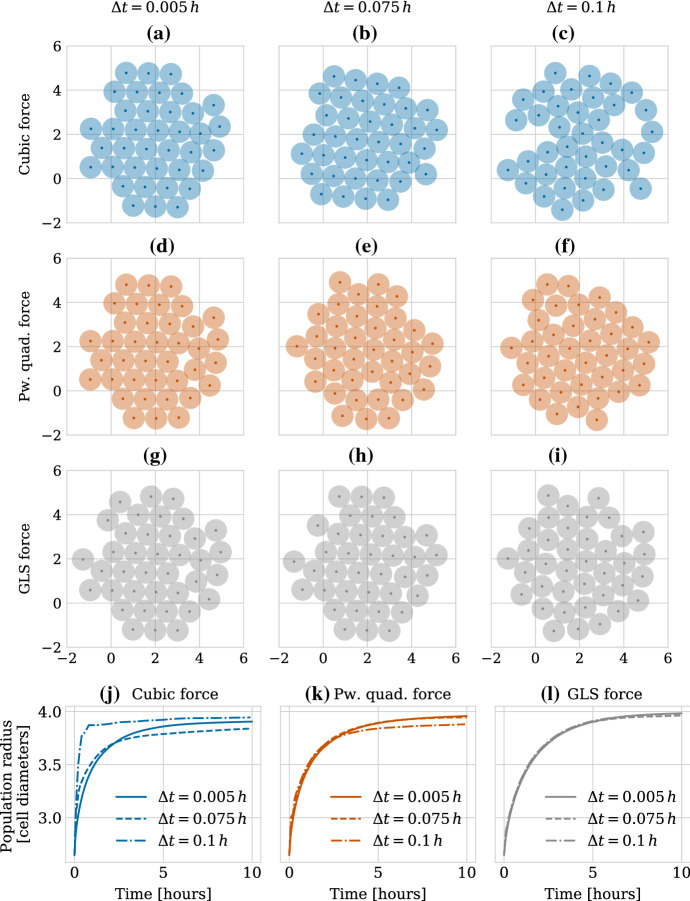
Impact of the time step size on the geometrical shape of a population and its radius. First row: 2D plots of a monolayer population simulated with the cubic force simulated for 10 h with the forward Euler method and different time step sizes, (**a**) $\varDelta t = 0.005\,h$, (**b**) $\varDelta t = 0.075\,h$ and (**c**) $\varDelta t = 0.1\,h$. Second row: same set-up simulated with the piecewise quadratic force. Third row: same set-up simulated with the GLS force. All time step values lie within the stability region of the forward Euler method for all three force functions. The last row shows the radius of a monolayer population over time. Initially the population consisted of 19 cells arranged in a regular honeycomb pattern. All cells divided simultaneously once at the beginning of the simulation (cell division vectors were consistent across time steps and force functions due to the random seed being fixed). Then, the population was allowed to relax to a mechanical equilibrium configuration. Results are averaged over 10 simulation runs. The force function used was (**j**) the cubic force, (**k**) the piecewise quadratic force and (**l**) the GLS force

The three upper rows of Fig. 12 show an example realisation of a monolayer population relaxing to a mechanical equilibrium after simultaneous cell divisions for different time steps under the three force functions. The leftmost column corresponds to an accurate solution at $t_\text {end} = 10\,h$, and the other two columns show the population for larger time step values at the same end time. We note that all chosen time steps lie within the stability bounds calculated for the pairwise relaxation case in the previous section, but not all lie within the monotonocity threshold (compare with Table 4). The random seed was fixed across time steps and force functions for this experiment, resulting in consistent cell division vectors. This means that all observed differences in the geometrical configurations are purely due to the accuracy of the numerical solution and to potential differences in the force functions.

As can be seen by comparing the simulations in the leftmost columns (Fig. 12a, d and g), we observe an almost identical positioning of the cells for the piecewise quadratic and GLS forces, with only slight differences from the cubic force for an accurately resolved simulation. To quantify this, the relative differences in centre positions between simulations using the GLS and the piecewise quadratic forces are $2.9\%$ and between the GLS and cubic forces $3.1\%$. These simulations were computed with a small time step $\varDelta t = 0.005\, h$, leading to a relative discretization error in centre positions of $0.5\%$ for the cubic force and slightly less for the piecewise quadratic ($\epsilon _\text {rel} = 0.4\%$) and the GLS forces ($\epsilon _\text {rel} = 0.3\%$). This illustrates that the parameterization strategy we have chosen, i.e. to match the pairwise relaxation time, also results in very similar dynamics even on the level of centre positions for initial stages of a two-dimensional monolayer growth given an accurate enough numerical solution to the equations of motion.

On the other hand, we observe relatively large geometrical deviations from the well-resolved case for all three force functions as we increase the time step sizes, but like in the pairwise case, the sensitivity depends a lot on the force function (Fig. 12c, f, i). Consistent with the fact that it had the strictest threshold for dynamics of pairwise relaxing cells, these geometrical differences are biggest for the cubic force function (Fig. 12a–c), and again, the GLS force is most robust to variations in the time step (Fig. 12g–i) with the piecewise quadratic force falling in between (Fig. 12d–f). This is more easily seen in Fig. 12j–l where we show the population radius as a function of time for different step sizes for each of the force functions. Again, the radius is most sensitive to the time step under the cubic force.

In summary, the experiment in this section illustrates that the consequences on population level behaviour can be substantial if the time step is not chosen with care. Again, we emphasize that for an accurate numerical solution (leftmost column), simulations with all three force functions—if appropriately parameterized—result in geometrically very close solutions, while the sensitivity to the time step size differs substantially.

### Convergence Study for First- and Second-Order Explicit Methods

In order to quantify the effect of the time step, we conducted a convergence study for each of the three experimental set-ups we have considered until now: (i) relaxation of overlapping cells after cell division simulated until $T=1\,h$, (ii) pairwise dynamics of closely adhering cells, initially placed at a distance of 1.15 cell diameters and simulated until mechanical relaxation at $T=3\,h$ and (iii) mechanical relaxation of two small proliferating monolayers of initially 19 and 37 cells simulated until $T=4\,h$. We considered the forward Euler method, the midpoint method and the Adams–Bashforth method. The forward Euler method—used in the majority of CBM software—is first order, as confirmed by the convergence graphs. The midpoint and the Adam-Bashforth methods are commonly used second-order schemes, where the latter is also the default solver in the PhysiCell software (Ghaffarizadeh et al. 2018).

**Fig. 13:**
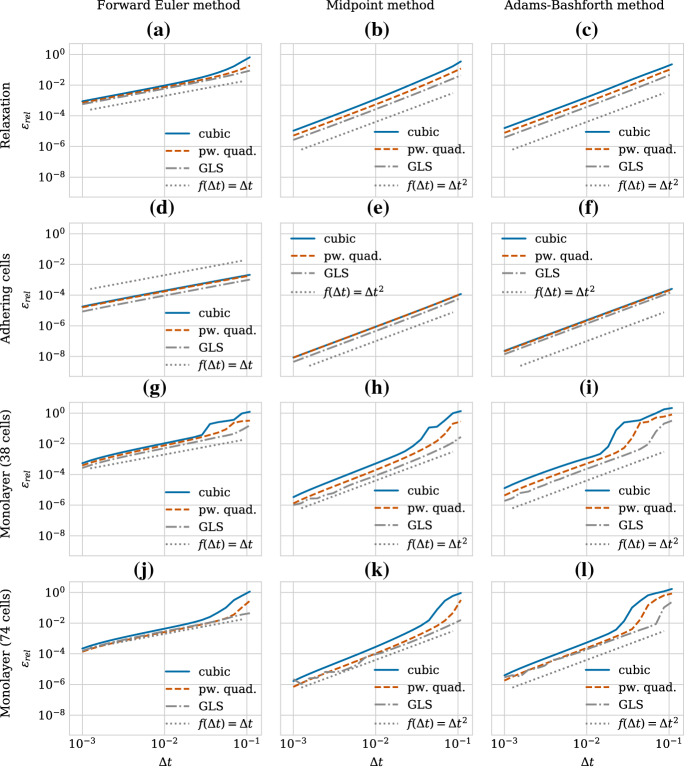
Impact of time step size on the relative error for the different combinations of forces and numerical solvers tested on the three model problems (i)–(iii). (**a**–**c**) Convergence study for the relaxation experiment (i), initial separation 0.3 cell diameters, (**d**–**f**) convergence study for adhering cells (ii), initial separation 1.15 cell diameters, (**g**–**i**) convergence study for a monolayer population of 38 (initially 19) cells and (**j**–**l**) convergence study for a monolayer population of 74 (initially 37) cells (iii). The numerical solver used was (**a**, **d**, **g**, **j**) the forward Euler method, (**b**, **e**, **h**, **k**) the midpoint method and (**c**, **f**, **i**, **l**) the Adams–Bashforth method. The axes of the plots are in logarithmic scale. The dotted lines show a linear (for plots (**a**, **d**, **g**, **j**)) and a quadratic function (all other plots) to facilitate observation of the convergence order

Figure 13 shows the relative error in the numerical solution as a function of time step, force function and numerical scheme in log-log plots. For a discrete time step $\varDelta t$, we computed the relative error as

20 $$\begin{aligned} \epsilon _{\text {rel}} = \frac{\Vert \mathbf{y} _{\varDelta t} -\mathbf{y} _\text {ref}\Vert _2}{\Vert \mathbf{y} _\text {ref}\Vert _2}. \end{aligned}$$

Here, $\mathbf{y} _{\text {ref}}$ is a gold standard reference solution computed with a very small time step for the respective force function. We used $\varDelta t_\text {ref} = 1\times 10^{-5}\,h$ for case (i), $\varDelta t_\text {ref} = 1\times 10^{-4}\,h$ for case (ii), and $\varDelta t_\text {ref} = 5\times 10^{-4}\,h$ for case (iii). We interpolated the coarser solution $\mathbf{y} _{\varDelta t}$ down to the fine time grid used for $\mathbf{y} _{\text {ref}}$.

As can be seen in Fig. 13, all three tested schemes show the expected convergence rate—first-order for the forward Euler method and second-order for the midpoint and the Adams–Bashforth methods—for all three test cases, i.e. for the pairwise relaxation case (Fig. 13a–c), for relaxation in the adhesive regime (Fig. 13d–f) and for the centre positions in two monolayers of different sizes (Fig. 13g–i, j–l), provided $\varDelta t$ is chosen small enough. The errors are larger overall for the repulsive part than for the adhesive one. This means that if we choose a time step that accurately resolves the transient repulsive regime we do not need to worry about the adhesive regime. This holds for all solvers and all force functions.

From the convergence study, we can draw some conclusions about the computational efficiency associated with the numerical methods and force functions. As can be seen, both second-order methods allow for approximately 4–$5\times$ larger time steps than the forward Euler method at a relative error level of $1\%$, and up to approximately $10\times$ at $0.1\%$. This is a substantial difference that is likely to lead to computational efficiency improvements in a state-of-the art implementation, even if two force function evaluations are needed at each time step rather than a single one for the forward Euler method. The midpoint method shows slightly better properties than the Adams–Bashforth method for large time steps. For this parameterization of the force functions, which results in close two-dimensional monolayer dynamics, the GLS force allows for up to 3–$4\times$ larger time steps with all solvers compared to the piecewise quadratic force, which in turn allows for $2\times$ larger time steps than cubic. This is consistent with the observations in the previous section and is a consequence of the faster asymptotic drop-off in force for the cubic force (it behaves more rigidly than the piecewise quadratic force which in turn leads to more rigid cells than GLS).

In summary, assuming we want the relaxation dynamics after cell division resolved to a relative error of $1\%$ in our simulation, the difference in time step between choosing the GLS force function and the midpoint method versus the cubic force and forward Euler method is an order of magnitude in our experiments. However, it will lead to almost identical simulations of the radius of a growing monolayer. This highlights the fact that substantial computational savings can be expected if force and numerical method are chosen carefully.

We note here that there are of course other differences between the force functions that might affect a modellers choice, such as the degree of flexibility with which repulsion and adhesion can be chosen independently of each other. We also note that it is in general hard or impossible to *a priori* relate the error for the simple pairwise case to more complex summary statistics of simulations of large, complex systems. For this reason, a good recommendation for choosing the time step *a priori* is to choose it such that the error in pairwise relaxation is small, since it is likely that the case of two cells undergoing transient relaxation from a highly compressed state represents the worst-case behaviour in a complex tissue simulation since there are no balancing, opposite forces from surrounding cells.

**Fig. 14:**
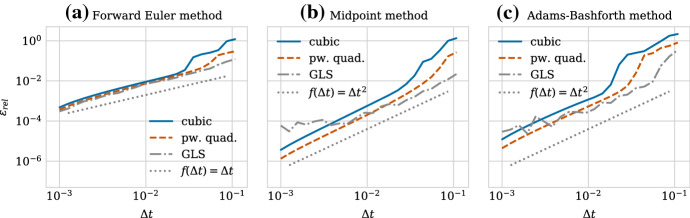
Convergence study for a monolayer population consisting initially of 19 cells. The parameter $\alpha$ of the GLS force was chosen independently of the spring stiffness $\mu$ as $\alpha = 7.51$ by fitting force intensities over the adhesive regime (strategy #1 in Sect. 3.2.1). The numerical solver used was (**a**) the forward Euler method, (**b**) the midpoint method and (**c**) the Adams–Bashforth method. The axes of the plots are in logarithmic scale. The dotted lines show a linear (plot (**a**)) and a quadratic function (plots (**b**) and (**c**)) to facilitate observation of the convergence order.

From a pure numerical robustness point of view, the GLS force shows some advantages over the piecewise quadratic and the cubic force in our experiments; however, here there are situations that need to be treated carefully. Recall that after comparing the effect of two different strategies for choosing its parameter $\alpha$ on the transient dynamics of adhering cells and their impact on the population-level behaviour in Sect. 3.2, we had chosen $\alpha$ such that we ensure a smooth drop-off to zero in force magnitude at the adhesive cut-off $r_A$ (strategy #2), as this provided a better agreement of the population radius. Figure 14 shows the convergence of the forward Euler, the midpoint and the Adams–Bashforth methods for the GLS force when we instead chose $\alpha$ independently of the spring stiffness parameter $\mu$ as $\alpha =7.51$ according to strategy #1. As can be seen, this choice of parameter leads to a loss of second-order convergence for both the midpoint and the Adams–Bashforth methods. This is because of the discontinuity in the force at the maximum interaction distance $r_A$. This illustrates that in particular when considering the use of higher-order methods, care needs to be taken to assure sufficient smoothness of the force function. The higher the order of the method, the more smoothness is needed.

### Comparison of Execution Times for First- and Second-Order Explicit Methods

**Fig. 15:**
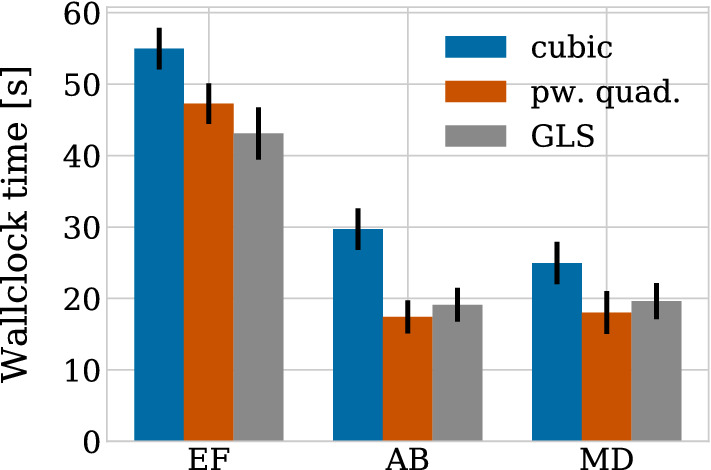
Average execution time for a relaxing monolayer initially consisting of 400 cells for a relative accuracy of $\epsilon _\text {rel} =10^{-3}$ for the different combinations of forces and numerical solvers. All cells divided at the beginning of the simulation. The monolayer then relaxed to mechanical equilibrium until time $t_\text {final}=4\,h$. EF—the forward Euler method, MD—the midpoint method, AB—the Adams–Bashforth method. One hundred simulations were run for each case and the error bars are one standard deviation around the mean value

To summarize our findings from the previous section, we should in general be able to expect computational efficiency gains of using a second-order solver such as the midpoint or the Adams–Bashforth method over the forward Euler method (unless we allow for quite large errors in the pairwise relaxation dynamics (around $10\%$ or higher)). Their second-order convergence means that this advantage grows the more well resolved we want our simulation to be. To get a feel for the gain, we lastly compared execution times for first- and second-order solvers. Figure 15 shows the wall clock time for the simulation of a monolayer of 400 cells for the different combinations of forces and numerical solvers. As in our other experiments, all cells divided simultaneously at the beginning of the simulation. The time step sizes were chosen to satisfy a relative accuracy of $\epsilon _\text {rel} =10^{-3}$ and are stated in Table 5 along with the number of steps of that size necessary to simulate the mechanical relaxation of the monolayer for 4 h in-simulation time. We observe significant gains of using the second-order midpoint and Adams–Bashforth methods for all three force functions compared to using the forward Euler method. For example, using the piecewise quadratic force and the midpoint method required less than 20 seconds, whereas using the same force and the forward Euler method took more than 40 seconds, i.e. double the time. Again, the cubic force performed slightly worse than the GLS and the piecewise quadratic forces, due to needing smaller time steps to resolve to the same accuracy (compare with Table 5). We stress that these gains can be expected to be even larger for a high-performance implementation.

**Table 5:** Time step $\varDelta t$ and number of steps *N* required for resolving the trajectories of a monolayer of 400 cells until time $t_\text {final}=4\,h$ for a relative accuracy of $\epsilon _\text {rel} =10^{-3}$ for the different combinations of forces and numerical solvers

	Forward Euler	Midpoint	Adams–Bashforth
Cubic force	$\varDelta t=0.0050$, $N=800$	$\varDelta t=0.0238$, $N=169$	$\varDelta t=0.0191$, $N=210$
Pw. quad. force	$\varDelta t=0.0063$, $N=635$	$\varDelta t=0.0373$, $N=108$	$\varDelta t=0.0373$, $N=108$
GLS force	$\varDelta t=0.0122$, $N=328$	$\varDelta t=0.0582$, $N=69$	$\varDelta t=0.0582$, $N=69$

## Discussion and Conclusion

In this paper, we have systematically studied to what extent the choice of the pairwise interaction force in CBM OS simulations affects the numerical robustness and accuracy of simulations. We have also introduced an empirical way of calibrating different forces to give a similar population-level behaviour for two-dimensional monolayer growth where repulsive interactions between cells dominate over adhesive ones.

The motivation for our study was twofold. Firstly, when engaging in modelling projects using open-source tools such as MecaGen, PhysiCell and Chaste, the question naturally arises to what extent the different default forces used—cubic, piecewise quadratic or GLS—will lead to any significant difference in the results. We focused on these three forces, because in our opinion they are those a modeller new to the field might come in contact with most easily. Secondly, given the assumption that the force functions are interchangeable from a modelling perspective, we were interested in whether their detailed form and implementation could have a significant impact on the simulation efficiency.

To study these questions, we considered the simplest possible implementation of a centre-based model (as described in Sect. 2). All code, tests and Jupyter Notebooks necessary to reproduce our study are available on GitHub. There are many more physically realistic and intricate implementations of neighbourhood definitions, the cell cycle and cell proliferation. However, this simple formulation is still widely adopted since it is most computationally efficient. This is important for a CBM because the target cell counts in simulations can be in the millions or higher, at least in the case of high-performance and parallel implementations. Our study focused on how the force function choice and the numerical solver affect the total number of time steps necessary to achieve a given accuracy in cell trajectories. Therefore, we did not aim to quantify nor optimise the performance per individual time step (e.g. we did not implement bounding boxes as discussed in Sect. 2.3), since such per-time-step optimisations would have benefitted all our studied configurations equally. Instead, the implementation was optimised to allow easy control of the numerical aspects of the simulation.

To be able to quantitatively compare numerical properties we focused on two model systems. The first was the pairwise relaxation dynamics following the placement of two cells with an overlap $r_0$, as considered similarly in the supplementary information of the PhysiCell software (Ghaffarizadeh et al. 2018). The second was the mechanical relaxation of a two-dimensional monolayer after intense proliferation. By asking that all three force functions we considered should result in the same (or rather very close) population radius, we could parameterize all forces to agree qualitatively. Interestingly, matching the pairwise relaxation times resulted in almost the same population-level dynamics for two different monolayer sizes. Future work should check if this still holds true for spheroid growth in three dimensions. Based on this parameterization, we could conclude that from a purely numerical efficiency point of view, there can be significant performance differences between the studied force functions if the objective is to simulate the same population-level metric.

The above parameterization based on the pairwise relaxation time did not determine the $\alpha$ parameter of the GLS force due to this force having two different functional forms in the repulsive and the adhesive regime. Instead, we compared two sub-parameterization strategies for $\alpha$. In the first, strategy #1, we fit the GLS force in intensity to the cubic force over the adhesive regime. In the second, strategy #2, we chose $\alpha$ depending on the spring stiffness such that the discontinuity of the GLS force was small at the maximum interaction distance. Both strategies resulted in qualitatively different transient dynamics for adhering cells (see Fig. 6). Interestingly, when studying how the fit to relaxation time transferred to the population level behaviour, the agreement in population radius was better for strategy #2. Future work should study if this holds true for more dispersive model systems where adhesive interactions dominate over repulsive forces. Moreover, the choice of strategy #2 also led to better numerical properties, see Fig. 14 and the discussion below.

An important assumption considered in the present work concerns the orientation of cell division. In all our simulations, the axis crossing the centres of two daughter cells upon cell division was chosen randomly. In some biological situations, however, cells tend to divide according to a specific direction. This mechanism plays a vital role in several biological processes, and especially in morphogenesis (Wyngaarden et al. 2010; Kaucka et al. 2017). There exist several computational models belonging to the cell-based type which study the mechanisms underlying oriented cell division and their role in embryogenesis and morphogenesis (Delile et al. 2017; Gord et al. 2014). These models show that the tissue elongates in the direction in which cells tend to divide. We did not study the impact of the force function formulation on this specific question because it was out of the scope of the present work. However, we expect that choosing large time steps would significantly increase the extent to which the tissue will be elongated.

Our study complements both the literature on comparisons of different types of CBMs (Osborne et al. 2017; Pathmanathan et al. 2009) and other careful evaluations of numerics for one particular force function (Ghaffarizadeh et al. 2018). One central issue we focused on is the possible overshoot for too large time steps for highly compressed cells right after cell division due to unphysically large force magnitudes. This problem is well known in the CBM methods developer community and different ways of dealing with it have been suggested (Van Liedekerke et al. 2018; Schaller and Meyer-Hermann 2005). Since the solution does not necessarily become numerically unstable and obviously wrong (see Fig. 12), there is a real risk for misinterpretations of results, not only quantitatively (which would be less of an issue given the large uncertainties in the model and the many model assumptions), but also qualitatively. An example of this are the clear differences in the visual appearance of the geometry, for example, a more loose structure with diffuse borders in Fig. 12c) caused by time steps violating the monotonicity threshold. There is a risk for attributing this difference to the physical parameters of the model. We contributed here with a quantitative, numerical analysis of this problem for the model problem of two cells relaxing right after cell division. In particular, we show how to compute limits on the time step to ensure a monotone solution without overshoot and compare such limits for the three force functions under study. Again, we emphasize that even for the forward Euler method—the dominating scheme used in implementations to solve the functions of motion—these limits are accuracy-based and not based on the formal numerical stability of the solution. In fact, for the forward Euler method this monotonicity bound is half as large as the stability bound.

In comparing errors and convergence with two commonly used second-order schemes, we found it likely that second-order schemes, such as the Adams–Bashforth method used in PhysiCell (Ghaffarizadeh et al. 2018) should provide a computational gain in high-performance implementations even if they require a more expensive update step. The gain becomes larger and larger the more accurately cell trajectories need to be resolved but outperforms the forward Euler method in our experiments even for modest accuracy requirements. Of the forces we considered, the GLS force used in Chaste (Cooper et al. 2020) has the best numerical robustness properties and leads to the lowest discretization errors, both for first- and second-order solvers. The cubic force function was most sensitive to changes in numerical parameters and required overall smaller time steps than both the piecewise quadratic and the GLS forces.

However, as seen in Sect. 4.3, care needs to be taken with the GLS force to choose parameters to preserve second-order convergence for second-order schemes. In general, sufficient smoothness of the force is a requirement for higher-order convergence and would likely become an issue also for the piecewise polynomial force for e.g. Runge–Kutta schemes unless the degree of the polynomial is chosen to match the order of the scheme (Ghaffarizadeh et al. 2018). We also note here that the challenge is to resolve the transient solution right after cell division and while a higher-order scheme allows for larger time steps than the forward Euler method, another possible approach to improve efficiency is time step adaptivity (Schaller and Meyer-Hermann 2005; Van Liedekerke et al. 2018; Atwell 2016). In such a setting it could be interesting to consider switching between implicit and explicit schemes during the course of a simulation. In general, however, it is unlikely in our opinion that using (only) higher-order implicit schemes would be beneficial for large systems, due to the substantial increase in cost per time step. One way to think about this is that we show (for the examples that we consider) that the time step is not necessarily governed by the numerical stability of the scheme. Instead accuracy requirements and requirements posed by maintaining a physically realistic solution force time steps to be smaller than the stability bound. This suggests that the potential benefits of using implicit schemes might not be realized.

To conclude, it is not possible to make a firm statement about what combination of force function and solver to use as a default based only on our study, since there are both qualitative and quantitative aspects to that question. For example, from a qualitative point of view, the piecewise quadratic force used in PhysiCell (Ghaffarizadeh et al. 2018) permits greater flexibility to vary the repulsive and adhesive spring stiffness parameters independently of each other, which is a very good feature for parametric studies. Other physics-based force functions not studied here such as the extended Hertz force (Drasdo et al. 2007) have the advantage of experimentally measurable parameters. The GLS force is the least flexible when it comes to the ability to be tuned to match dynamics of other force functions and it changes functional form in the adhesive regime. Finally, there are many important considerations when choosing a force function, such as physical realism, modelling flexibility, ease of implementation, etc. However, given a specification of those requirements, we demonstrated here that the difference in time steps for a given accuracy could easily be as big as an order of magnitude for a highly resolved simulation purely dependent on the specifics of the force choice.
